# Acute effects of *R*-MDMA, *S*-MDMA, and racemic MDMA in a randomized double-blind cross-over trial in healthy participants

**DOI:** 10.1038/s41386-024-01972-6

**Published:** 2024-08-23

**Authors:** Isabelle Straumann, Isidora Avedisian, Aaron Klaiber, Nimmy Varghese, Anne Eckert, Deborah Rudin, Dino Luethi, Matthias E. Liechti

**Affiliations:** 1 Clinical Pharmacology and Toxicology, Department of Biomedicine and Department of Clinical Research, University Hospital Basel, Basel, Switzerland; 2 Department of Pharmaceutical Sciences, University of Basel, Basel, Switzerland; 3 Psychiatric University Hospital, University of Basel, Basel, Switzerland; 4 Transfaculty Research Platform Molecular and Cognitive Neuroscience, University of Basel, Basel, Switzerland

**Keywords:** Pharmacology, Translational research

## Abstract

Racemic 3,4-methylenedioxymethamphetamine (MDMA) acutely increases mood, feelings of empathy, trust, and closeness to others and is investigated to assist psychotherapy. Preclinical research indicates that *S*-MDMA releases monoamines and oxytocin more potently than *R*-MDMA, whereas *R*-MDMA more potently stimulates serotonin 5-hydroxytryptamine-2A receptors. *S*-MDMA may have more stimulant properties, and *R*-MDMA may be more psychedelic-like. However, acute effects of *S*- and *R*-MDMA have not been examined in a controlled human study. We used a double-blind, randomized, placebo-controlled, crossover design to compare acute effects of MDMA (125 mg), *S*-MDMA (125 mg), *R*-MDMA (125 mg and 250 mg), and placebo in 24 healthy participants. Outcome measures included subjective, autonomic, and adverse effects, pharmacokinetics, and plasma oxytocin, prolactin, and cortisol concentrations. *S*-MDMA (125 mg) induced greater subjective effects (“stimulation,” “drug high,” “happy,” “open”) and higher increases in blood pressure than *R*-MDMA (both 125 and 250 mg) and MDMA (125 mg). Unexpectedly, *R*-MDMA did not produce more psychedelic-like effects than *S*-MDMA. *S*-MDMA increased plasma prolactin more than MDMA, and *S*-MDMA increased plasma cortisol and oxytocin more than MDMA and *R*-MDMA. The plasma elimination half-life of *S*-MDMA was 4.1 h after administration. The half-life of *R*-MDMA was 12 and 14 h after the administration of 125 and 250 mg, respectively. Half-lives for *S*-MDMA and *R*-MDMA were 5.1 h and 11 h, respectively, after racemic MDMA administration. Concentrations of the CYP2D6-formed MDMA-metabolite 4-hydroxy-3-methoxymethamphetamine were lower after *R*-MDMA administration compared with *S*-MDMA administration. The pharmacokinetic findings are consistent with the *R*-MDMA-mediated inhibition of CYP2D6. Stronger stimulant-like effects of *S*-MDMA in the present study may reflect the higher potency of *S*-MDMA rather than qualitative differences between *S*-MDMA and *R*-MDMA. Equivalent acute effects of *S*-MDMA, MDMA, and *R*-MDMA can be expected at doses of 100, 125, and 300 mg, respectively, and need to be investigated.

Trial registration: ClinicalTrials.gov identifier: NCT05277636

## Introduction

3,4-Methylenedioxymethamphetamine (MDMA) releases serotonin (5-hydroxytryptamine [5-HT]), norepinephrine, dopamine, and oxytocin and induces feelings of well-being, empathy, trust, closeness, and connectedness [1, 2]. Acute subjective effects of MDMA are considered helpful to assist psychotherapy for posttraumatic stress disorder [3]. MDMA is a racemic substance that contains equal amounts of the enantiomers *S*(+)- and *R*(-)-MDMA. Preclinical research indicates that *S*-MDMA more potently releases monoamines and oxytocin than *R*-MDMA, whereas *R*-MDMA may act more potently on 5-HT_2A_ receptors [4–10]. Behavioral animal studies indicate that *S*-MDMA is more stimulant-like than *R*-MDMA, and *R*-MDMA may be more psychedelic-like while still producing MDMA-typical effects [11–13]. For example, the stimulant d-amphetamine substituted for *S*-MDMA- but not *R*-MDMA-trained animals while the psychedelic 2,5-dimethoxy-4-propylthiophenethylamine substituted for *R*-MDMA- but not *S*-MDMA-trained animals in drug-discrimination studies in mice [11]. Additionally, preclinical research indicates that *R*-MDMA induces less hyperthermia and less neurotoxicity [14–16]. Research on abuse-related behavioral effects in Rhesus monkeys showed comparable [17] or little to no drug self-administration of *R*-MDMA compared with MDMA and *S*-MDMA [18]. Consistently, priming with MDMA or *S*-MDMA but not with *R*-MDMA reinstated extinguished amphetamine self-administration behavior [19]. Because of these preclinical results, *R*-MDMA has been discussed as a potentially safer tool for substance-assisted therapy than racemic MDMA [12]. However, acute effects of *S*- and *R*-MDMA have not been validly compared in a human study. Therefore, the present study compared acute responses to racemic MDMA, *S*-MDMA, *R*-MDMA, and placebo in a double-blind, crossover study in healthy participants.

The primary study hypothesis was that *S*-MDMA would induce greater ratings of subjective stimulation on the Visual Analog Scale (VAS) than *R*-MDMA, and *R*-MDMA would induce more psychedelic-like effects on the 5 Dimensions of Altered States of Consciousness (5D-ASC) scale than *S*-MDMA.

## Methods and materials

### Study design

The study used a double-blind, placebo-controlled, crossover design with five experimental test sessions to investigate responses to (*i*) placebo, (*ii*) 125 mg racemic MDMA, (*iii*) 125 mg *S*-MDMA, (*iv*) 125 mg *R*-MDMA, and (*v*) 250 mg *R*-MDMA. Participants were informed that they would get all treatments. Block randomization was used with counterbalanced treatment order. The washout periods between sessions were at least 10 days. The study was conducted in accordance with the Declaration of Helsinki and International Conference on Harmonization Guidelines in Good Clinical Practice and approved by the Ethics Committee of Northwest Switzerland (EKNZ) and Swiss Federal Office for Public Health. The study was registered at ClinicalTrials.gov (NCT05277636).

### Participants

Twenty-four healthy participants (12 men and 12 women; mean age ±SD: 29 ± 9 years; range: 18–47 years) were recruited by word of mouth or from a pool of volunteers who had contacted our research group because they were interested in participating in a clinical trial on psychedelics or entactogens. All of the subjects provided written informed consent and were paid for their participation. Exclusion criteria were <18 years or >65 years of age, pregnancy (urine pregnancy test at screening and before each test session), personal or family (first-degree relative) history of major psychiatric disorders (assessed by the Semi-structured Clinical Interview for *Diagnostic and Statistical Manual of Mental Disorders*, 5th edition, Axis I disorders), the use of medications (e.g., antidepressants, antipsychotics, and sedatives) that may interfere with the study medications, chronic or acute physical illness (e.g., abnormal physical exam, electrocardiogram, or hematological and chemical blood analyses), tobacco smoking (>10 cigarettes/day), lifetime prevalence of illicit substances >20 times or use within the last 2 months (except for Δ^9^-tetrahydrocannabinol; THC), and illicit drug use during the study period (including THC; urine drug test performed randomly prior to one study day). The participants were asked to consume no more than 15 standard alcoholic drinks/week and have no more than one drink on the day before the test sessions. Prior and current substance use is described in the Supplementary Methods and in Supplementary Table S1.

### Study drugs

We selected 125 mg racemic MDMA as a common and safe dose [20]. Based on animal data, 125 mg *S*-MDMA and 125 mg racemic MDMA were expected to be overall equipotent in inducing stimulant-type and adverse effects in humans. *R*-MDMA was administered at a dose of 125 mg and additionally at a higher dose of 250 mg based on its lower potency and to be able to assess its effect characteristics more fully. Preliminary data indicated that *S*-MDMA was active at 80–120 mg, and *R*-MDMA was expected to be active at doses near 300 mg in humans [21]. Fixed rather than weight-based doses were used for practical reasons and because MDMA has not been adjusted to body weight in phase 3 studies and in limited use outside clinical studies. MDMA (ReseaChem, Burgdorf, Switzerland) was administered in opaque capsules that contained 25 mg MDMA hydrochloride and an exact analytically confirmed actual MDMA content of 25.40 ± 0.48 mg (*n* = 9 samples). *S*-MDMA (ReseaChem, Burgdorf, Switzerland) was administered in opaque capsules that contained 25 mg *S*-MDMA hydrochloride and an exact analytically confirmed actual *S*-MDMA content of 25.56 ± 0.62 mg (*n* = 10). *R*-MDMA (ReseaChem, Burgdorf, Switzerland) was administered in opaque capsules that contained 25 mg *R*-MDMA hydrochloride and an exact analytically confirmed actual *R*-MDMA content of 25.50 ± 1.30 mg (*n* = 10). Placebo consisted of identical opaque capsules that were filled with mannitol. All capsules were produced according to Good Manufacturing Practice guidelines (Dr. Hysek AG, Biel, Switzerland). The subjects received 10 capsules in each session: (*i*) 10 placebo capsules, (*ii*) five 25 mg (±)-MDMA capsules and five placebo capsules, (*iii*) five *S*-MDMA capsules and five placebo capsules, (*iv*) five 25 mg *R*-MDMA capsules and five placebo capsules, and (*v*) ten 25 mg *R*-MDMA capsules. At the end of each session and at the end of the study, the participants guessed their treatment assignment to evaluate blinding.

### Study procedures

The study included a screening visit, five 10-h test sessions with follow-up measurements 24 h after drug intake, and an end-of-study visit that occurred an average of 14 days after the last test session. The sessions were conducted in a calm hospital room. Only one research participant and one investigator were present during each test session. The test sessions began at 8:00 AM. A urine pregnancy test was performed in women with childbearing potential. The participants underwent baseline measurements. A standardized breakfast (two croissants) was served. Substances were administered at 9:00 AM. The outcome measures were repeatedly assessed for 9 h. Standardized lunches were served at 1:30 PM. The participants were sent home at 6:15 PM and returned the next day for follow-up measurements at 9:00 AM.

### Subjective drug effects and effect durations

Subjective effects were assessed repeatedly using VASs 0.5 h before and 0, 0.25, 0.5, 0.75, 1, 1.5, 2, 2.5, 3, 3.5, 4, 5, 6, 7, 8, 9, and 24 h after drug administration. The VAS “simulated” was the primary measure to assess stimulation. The Adjective Mood Rating Scale (AMRS) [22] was used 0.5 h before and 2.5, 5, and 9 h after drug administration. The 5D-ASC scale [23] and the 3D-ASC total score were used as the primary measure to assess psychedelic-like effects. It was administered 9 h after drug administration to retrospectively rate peak drug effects. Mystical experiences were assessed 9 h after drug administration using the Psychedelic Experience Scale (PES) [24], a revalidation of the 100-item States of Consciousness Questionnaire (SOCQ) [25], which includes the 30-item Mystical Experience Questionnaire (MEQ30) [24, 26]. Subjective effect measurements are described in detail in the Supplementary Methods online.

The time to onset, time to maximal effect, time to offset, effect duration, and area under the effect curve were assessed using Phoenix WinNonlin 8.3 (Certara, Princeton, NJ, USA) and “any drug effect” VAS effect-time plots and an onset/offset threshold of 10% of the maximum possible response. Participants with responses <10% on this scale were not used to determine the time to onset, time to offset, or effect duration.

### Autonomic and adverse effects

Blood pressure, heart rate, and tympanic body temperature were repeatedly measured at baseline and 0, 0.25, 0.5, 0.75, 1, 1.5, 2, 2.5, 3, 3.5, 4, 5, 6, 7, 8, 9, and 24 h after drug administration. Adverse effects were assessed 0.5 h before and 9, 24 and 72 h after drug administration using the List of Complaints [27]. To assess adverse effects on mood 1–3 days after substance administration, the Beck Depression Inventory (BDI) [28] and Symptom-Check-List-90-R (SCL-90-R) [29] were used 72 h after administration.

### Endocrine effects

Plasma concentrations of oxytocin were measured before and 2, 3, and 6 h after drug administration and determined as previously described [30]. Plasma concentrations of cortisol and prolactin were measured at baseline and 2 and 3 h after drug administration using an electrochemiluminescence immunoassay as previously described [31].

### Plasma MDMA concentrations

Plasma concentrations of MDMA, *S*-MDMA, *R*-MDMA, and their metabolites were measured before and 0.25, 0.5, 0.75, 1, 1.5, 2, 2.5, 3, 3.5, 4, 5, 6, 7, 8, 9, and 24 h after drug administration. Blood was collected into lithium heparin tubes. The blood samples were immediately centrifuged, and the plasma was subsequently stored at −80 °C until analysis.

MDMA, *S*-MDMA, *R*-MDMA, and their metabolites 3,4-methylenedioxyamphetamine (MDA) and 4-hydroxy-3-methoxymethamphetamine (HMMA) were analyzed in human plasma using an achiral high-performance liquid chromatography tandem mass spectrometry method and additionally an enantioselective method for racemic MDMA as previously described [32]. HMMA concentrations were determined after enzymatic deglucuronidation.

### Pharmacokinetic analyses

Pharmacokinetic parameters were estimated using non-compartmental methods. Analyses were conducted using Phoenix WinNonlin 8.3 (Certara, Princeton, NJ, USA).

### Data analysis

Peak (E_max_ and/or E_min_) or peak change from baseline (ΔE_max_) values were determined for repeated measures. The values were then analyzed using repeated-measures analysis of variance (ANOVA), with drug as the within-subjects factor, followed by the Tukey *post hoc* tests using R 4.2.1 software (RStudio, PBC, Boston, MA, USA). The criterion for significance was *p* < 0.05.

## Results

### Subjective drug effects

**Fig. 1:**
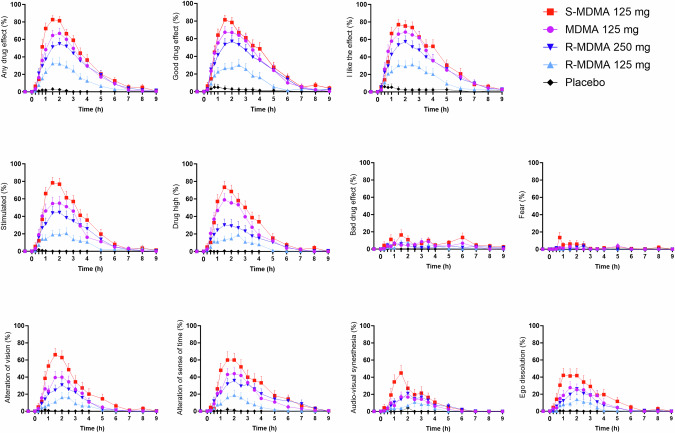
Acute subjective effects of 125 mg MDMA, 125 mg *S*-MDMA, 125 mg *R*-MDMA, and 250 mg *R*-MDMA on the Visual Analog Scale (VAS). *S*-MDMA produced overall stronger subjective responses than MDMA, with significant differences in “bad drug effects,” “alteration of vision,” and “audio-visual synesthesia.” *R*-MDMA at both doses produced overall lower subjective effects than MDMA, with significant differences in “drug high,” “happy,” “content,” “talkative,” “open,” “trust,” and “I feel close to others.” The substances were administered at *t* = 0 h. The data are expressed as the mean ± SEM percentage of maximally possible scores in 24 participants. The corresponding maximal responses and statistics are shown in Table 1.

**Fig. 2:**
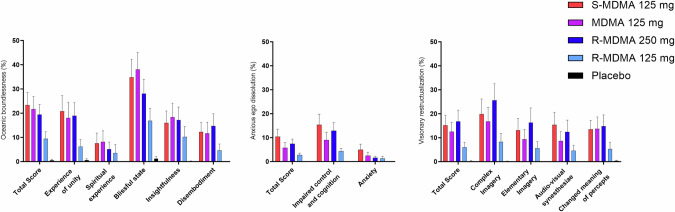
Acute mystical-type experiences on the 5 Dimensions of Altered States of Consciousness (5D-ASC) scale. MDMA, *S*-MDMA, and 250 mg *R*-MDMA induced comparable alterations of mind. The data are expressed as the mean ± SEM percentage of maximally possible scale scores in 24 participants. Statistics are shown in Supplementary Table S5.

Subjective effects over time on the VAS are shown in Fig. 1 and Supplementary Fig. S1. Subjective peak responses and statistics are shown in Table 1. *S*-MDMA produced overall greater subjective effects than MDMA and *R*-MDMA at the doses used. Specifically, *S*-MDMA induced significantly stronger “bad drug effects,” “alteration of vision,” and “audio-visual synesthesia” than MDMA and significantly stronger effects than 250 mg *R*-MDMA on most VASs including “stimulation”. Both *R*-MDMA doses induced lower effects on “drug high,” “happy,” “content,” “talkative,” “open,” “trust,” and “I feel close to others” than MDMA and *S*-MDMA (Fig. 1, Supplementary Fig. S1, Table 1). Responses in female participants were greater than in male participants due to lower body weights in women (Supplementary Fig. S2 and Supplementary Table S2). Responses in participants with and without previous MDMA experiences did not differ (Supplementary Fig. S3 and Supplementary Table S3). The mean effect duration was 3.5, 4.2, 4.7, and 5.2 h after the administration of 125 mg *R*-MDMA, MDMA, *S*-MDMA, and 250 mg *R*-MDMA, respectively (Supplementary Table S4). MDMA, *S*-MDMA, and 250 mg *R*-MDMA induced comparable alterations of mind and mystical-type effects on the 5D-ASC and PES48/MEQ, respectively (Fig. 2, Supplementary Fig. S4, statistics in Supplementary Tables S5 and S6). R-MDMA and S-MDMA also similarly increased the 3D-ASC total score reflecting comparable psychedelic effects (Supplementary Table S5). On the AMRS, 250 mg *R*-MDMA induced significantly higher “Introversion” than MDMA, and *S*-MDMA induced more “emotional excitation” than *R*-MDMA (Supplementary Fig. S5, Supplementary Table S7).

**Table 1:** Mean values and statistics for the acute subjective effects of MDMA, S-MDMA, R-MDMA and placebo.

		Placebo	125 mg R-MDMA	250 mg R-MDMA	125 mg MDMA	125 mg S-MDMA	F_4, 92_	*P*=	Pla - R-MDMA (125 mg)	Pla - R-MDMA (250 mg)	Pla - MDMA	Pla - S-MDMA	R-MDMA (125 mg) - R-MDMA (250 mg)	R-MDMA (125 mg) - MDMA	R-MDMA (125 mg) - S-MDMA	R-MDMA (250 mg) - MDMA	R-MDMA (250 mg) - S-MDMA	MDMA - S-MDMA
		Mean ± SEM	Mean ± SEM	Mean ± SEM	Mean ± SEM	Mean ± SEM												
Visual Analog Scale (VAS, %max)																		
Unidirectional scales (0–100)																		
Any drug effect	ΔE_max_	4.8 ±2.1	42 ±7.3	66 ±6.1	77 ±5.3	90 ±3.1	68.92	<0.001	***	***	***	***	***	***	***	NS	**	NS
Good drug effect	ΔE_max_	7.9 ±4.1	43 ±7.1	68 ±6.6	78 ±5.1	90 ±3.8	54.97	<0.001	***	***	***	***	***	***	***	NS	**	NS
Bad drug effect	ΔE_max_	0.5 ±0.3	14 ±5.1	19 ±4.6	20 ±5.9	39 ±7.0	10.51	<0.001	NS	*	*	***	NS	NS	***	NS	*	*
I like the effect	ΔE_max_	7.7 ±3.8	47 ±7.1	68 ±6.6	81 ±5.0	91 ±3.6	60.12	<0.001	***	***	***	***	**	***	***	NS	**	NS
Stimulated	ΔE_max_	2.5 ±1.1	31 ±6.9	60 ±6.7	70 ±6.7	88 ±3.7	58.82	<0.001	***	***	***	***	***	***	***	NS	***	NS
Drug high	ΔE_max_	1.4 ±0.6	29 ±7.0	48 ±7.6	73 ±6.7	84 ±5.1	49.31	<0.001	***	***	***	***	*	***	***	**	***	NS
Fear	ΔE_max_	0.0 ±0.0	4.9 ±4.2	5.8 ±3.4	5.9 ±3.4	19 ±6.9	3.90	0.006	NS	NS	NS	**	NS	NS	*	NS	NS	NS
Alteration of vision	ΔE_max_	3.0 ±1.6	24 ±6.9	37 ±6.7	54 ±7.8	74 ±6.7	32.23	<0.001	*	***	***	***	NS	***	***	NS	***	*
Alteration of sense of time	ΔE_max_	2.2 ±2.0	28 ±7.2	46 ±7.7	60 ±6.8	73 ±6.6	34.55	<0.001	**	***	***	***	NS	***	***	NS	**	NS
Audio-visual synesthesia	ΔE_max_	4.6 ±4.2	15 ±5.9	31 ±6.8	29 ±6.8	53 ±8.3	16.01	<0.001	NS	**	**	***	NS	NS	***	NS	**	**
Ego dissolution	ΔE_max_	1.5 ±0.8	22 ±7.4	32 ±7.4	41 ±7.7	58 ±8.2	17.83	<0.001	*	***	***	***	NS	NS	***	NS	**	NS
Bidirectional scales (−50 to 50)																		
Happy	ΔE_max_	3.9 ±2.2	14 ±3.5	24 ±3.8	34 ±3.3	38 ±3.4	32.25	<0.001	*	***	***	***	NS	***	***	*	***	NS
Content	ΔE_min_	4.8 ±2.2	19 ±3.4	30 ±3.6	39 ±2.8	44 ±2.5	46.83	<0.001	***	***	***	***	*	***	***	*	***	NS
Talkative	ΔE_max_	2.8 ±1.6	13 ±3.2	22 ±3.9	35 ±3.6	37 ±3.9	30.55	<0.001	NS	***	***	***	NS	***	***	**	**	NS
Open	ΔE_max_	4.3 ±2.3	15 ±3.4	28 ±3.9	40 ±2.9	42 ±2.9	50.64	<0.001	*	***	***	***	**	***	***	**	***	NS
Trust	ΔE_max_	4.0 ±2.0	16 ±3.5	24 ±4.1	40 ±3.1	44 ±2.3	48.93	<0.001	**	***	***	***	NS	***	***	***	***	NS
I feel close to others	ΔE_max_	0.8 ±0.4	14 ±3.1	23 ±3.9	34 ±3.3	41 ±3.4	43.83	<0.001	**	***	***	***	NS	***	***	**	***	NS
I want to be alone	ΔE_max_	0.2 ±0.2	6.1 ±2.6	10 ±3.4	6.9 ±2.7	12 ±3.4	4.15	0.004	NS	*	NS	**	NS	NS	NS	NS	NS	NS
I want to be with others	ΔE_max_	1.8 ±1.1	17 ±3.4	24 ±4.0	31 ±3.5	40 ±3.5	32.15	<0.001	***	***	***	***	NS	**	***	NS	***	NS
Autonomic effects																		
Systolic blood pressure (mmHg)	E_max_	127 ±2.7	138 ±2.5	147 ±2.5	152 ±2.3	160 ±3.1	74.46	<0.001	***	***	***	***	***	***	***	NS	***	**
Diastolic blood pressure (mmHg)	E_max_	78 ±0.8	84 ±1.1	91 ±1.6	92 ±1.3	97 ±1.6	62.90	<0.001	***	***	***	***	***	***	***	NS	***	***
Mean arterial pressure (mmHg)	E_max_	94 ±1.0	101 ±1.4	108 ±1.8	111 ±1.3	117 ±1.9	93.43	<0.001	***	***	***	***	***	***	***	NS	***	***
Heart rate (beats/min)	E_max_	75 ±1.3	87 ±2.6	93 ±3.4	95 ±3.5	100 ±3.3	21.50	<0.001	***	***	***	***	NS	NS	***	NS	NS	NS
Rate pressure product (mmHg x bpm)	E_max_	9135 ±218	11565 ±476	12999 ±569	14018 ±662	15389 ±705	41.15	<0.001	***	***	***	***	NS	***	***	NS	***	NS
Body temperature (°C)	E_max_	37.1 ±0.06	37.4 ±0.09	37.5 ±0.09	37.6 ±0.08	37.7 ±0.10	10.25	<0.001	NS	***	***	***	NS	NS	*	NS	NS	NS
List of complaints (LC score)																		
Acute adverse effects	0–9 h	0.5 ±0.5	9.4 ±1.8	14 ±1.9	10 ±1.7	13 ±1.5	21.58	<0.001	***	***	***	***	NS	NS	NS	NS	NS	NS
Subacute adverse effects	9–24 h	0.1 ±0.5	6.7 ±1.9	8.2 ±1.5	5.4 ±1.3	7.2 ±1.4	8.95	<0.001	***	***	**	***	NS	NS	NS	NS	NS	NS
Subacute adverse effects	24–72 h	1.3 ±0.7	6.9 ±1.8	6.0 ±1.7	5.1 ±1.6	10 ±2.2	6.05	<0.001	*	NS	NS	***	NS	NS	NS	NS	NS	NS
Becks depression-inventar (BDI)																		
BDI Score	24–72 h	2.0 ±0.9	4.3 ±1.2	4.4 ±1.2	4.8 ±1.3	8.4 ±1.7	4.07	0.004	NS	NS	NS	*	NS	NS	NS	NS	NS	NS
Symptom checklist 90 R (SCL-90-R)																		
GSI score	24–72 h	0.08 ±0.02	0.18 ±0.05	0.17 ±0.05	0.14 ±0.03	0.34 ±0.77	5.02	0.001	NS	NS	NS	*	NS	NS	NS	NS	NS	NS
Hormones and markers																		
Oxytocin (pg/mL)^α^	ΔC_max_	2.9 ±1.9	48 ±13	178 ±22	296 ±33	436 ±40	66.2^β^	<0.001	NS	***	***	***	***	***	***	**	***	***
Cortisol (nmol/L)	ΔC_max_	−156 ±27	140 ±28	249 ±26	285 ±26	398 ±27	81.90	<0.001	***	***	***	***	**	**	***	NS	***	*
Prolactin (µg/L)	ΔC_max_	−6.5 ±2.0	13 ±4.5	35 ±5.5	39 ±7.1	53 ±8.5	32.20	<0.001	***	***	***	***	***	**	***	NS	NS	**

*NS* not significant, *ΔE*_*max*_ maximal effect difference from baseline, *ΔC*_*max*_ maximal plasma concentrationfrom baseline, *N* = 24, *αN* = 20, β = F_4,81_.

*P < 0.05; **P < 0.01; ***P < 0.001.

### Autonomic and adverse effects

**Fig. 3:**
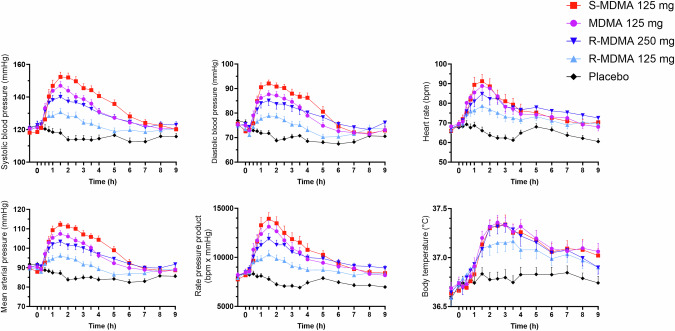
Acute autonomic effects. *S*-MDMA induced greater increases in blood pressure compared with MDMA and both *R*-MDMA doses. MDMA, *S*-MDMA, and 250 mg *R*-MDMA increased heart rate and body temperature comparably. The substances were administered at *t* = 0 h. The data are expressed mean ± SEM in 24 participants. The corresponding maximal responses and statistics are shown in Table 1.

Autonomic effects over time and related peak responses are shown in Fig. 3 and Table 1, respectively. *S*-MDMA induced higher increases in blood pressure than MDMA and *R*-MDMA. MDMA, *S*-MDMA, and 250 mg *R*-MDMA increased heart rate and body temperature comparably.

All substances produced similar acute and subacute adverse effects on the List of Complaints (Table 1). Frequently reported adverse effects included fatigue, headache, decreased appetite, feeling dull, lack of concentration, and dry mouth (Supplementary Table S8). All substances nominally increased self-ratings of depressive mood on the BDI 1–3 days after substance administration. Significantly higher ratings were seen for *S*-MDMA compared with placebo, with no significant differences between active drug substances (Table 1). No severe adverse events were observed.

### Endocrine effects

All substances increased plasma prolactin and cortisol compared with placebo. *S*-MDMA increased plasma prolactin more than MDMA and plasma oxytocin and cortisol more than MDMA and *R*-MDMA (Supplementary Fig. S6, Table 1).

### Plasma drug concentrations

Pharmacokinetic parameters are shown in Table 2. Concentration-time curves are shown in Supplementary Figs. S7–S9. Elimination half-lives (t_1/2_) for *S*-MDMA and *R*-MDMA were 5.1 and 11 h, respectively, when racemic MDMA was administered. The half-life of *S*-MDMA was 4.1 h when it was administered alone. The half-life of *R*-MDMA was 12 and 14 h after administration of the 125 and 250 mg doses, respectively (Table 2).

**Table 2:** Pharmacokinetic parameters based on non-compartmental analyses [geometric mean (95% CI), range], *N* = 24.

	C_max_ (ng/mL)	*t*_max_ (h)	*t*_1/2_ (h)	AUC_24_ (ng·h/mL)	AUC_∞_ (ng·h/mL)	CL/F (L/h)	V_z_/F (L)
125 mg (±)-MDMA							
(±)-MDMA	290 (263–320)	2.9 (2.5–3.5)	8.7 (7.6–10)	3274 (2881–3722)	4007 (3390–4738)	31 (26–37)	392 (364–422)
	180–408	1.5–7.0	4.6–16	1659–5209	1735–7591	16–72	273–530
(±)-MDA	14 (12–17)	6.8 (5.9–7.7)	14 (11–18)	231 (197–271)	340 (231–500)	368 (250–540)	7492 (6114–9181)
	6.8–28	3.0–9.0	8.0–29	95–455	112–1145	109–1115	4376–12,862
(±)-HMMA	141 (112–177)	2.9 (2.5–3.4)	12 (11–13)	1666 (1332–2084)	2274 (1814–2851)	55 (44–69)	943 (750–1186)
	49–431	1.5–7.0	7.7–18	563–4876	648–6070	21–193	304–2456
S-MDMA	123 (111–137)	2.8 (2.4–3.4)	5.1 (4.7–5.5)	1051 (933–1186)	1111 (977–1263)	56 (49–64)	413 (379–450)
	72–189	1.5–7.0	3.5–7.4	567–1571	574–1710	37–109	292–595
S-MDA	12 (10–14)	6.3 (5.5–7.3)	11 (9.3–13)	158 (128–197)	230 (187–283)	272 (221–334)	4311 (3632–5119)
	5.8–24	3.0–9.0	7.0–17	37–315	144–449	139–433	2294–6726
R-MDMA	167 (151–184)	3.3 (2.7–4.1)	11 (9.1–13)	2224 (1944–2544)	2995 (2436–3681)	21 (17–26)	327 (301–356)
	100–232	2.0–9.0	5.1–24	1092–3539	1166–6410	9.8–54	231–456
R-MDA	4.2 (3.6–5.0)	14 (11–18)		72 (58–89)			
	2.4–11	7.0–24		13–171			
125 mg S-MDMA							
S-MDMA	239 (215–265)	2.8 (2.3–3.4)	4.1 (3.6–4.6)	1869 (1659–2106)	1917 (1680–2187)	65 (57–74)	382 (349–418)
	137–413	1.0–8.0	2.3–7.4	949–2862	954–3051	41–131	253–606
S-MDA	21 (18–25)	5.6 (5.0–6.3)	8.0 (6.9–9.2)	261 (209–326)	349 (296–411)	358 (304–422)	4126 (3443–4944)
	9.0–46	3.0–9.0	4.0–12	62–608	196–761	164–637	2045–8142
HMMA	175 (145–211)	3.6 (3.2–4.1)	7.7 (6.8–8.8)	1955 (1666–2294)	2293 (1973–2665)	55 (47–63)	609 (491–754)
	71–421	1.5–7.0	4.8–13	822–3581	989–3768	33–126	249–1564
125 mg R-MDMA							
R-MDMA	335 (305–368)	3.2 (2.7–3.8)	12 (11–14)	4775 (4249–5366)	6869 (5803–8132)	18 (15–22)	328 (298–361)
	209–463	2.0–7.0	6.6–32	2307–6916	2559–14771	8.5–49	199–550
R-MDA	8.2 (6.8–9.8)	16 (13–20)		146 (121–175)			
	3.4–18	7.0–24		61–337			
HMMA	142 (105–191)	2.3 (1.8–2.8)	19 (17–22)	1631 (1300–2045)	2956 (2369–3688)	42 (34–53)	1181 (943–1479)
	28–551	0.8–6.0	12–39	500–5026	730–7100	18–171	376–4297
250 mg R-MDMA							
R-MDMA	694 (638–755)	3.6 (2.9–4.3)	14 (13–16)	10,087 (9113–11,164)	15754 (13,939–17,805)	16 (14–18)	329 (302–358)
	501–975	1.5–8.0	10–28	5770–15,780	10,049–29,136	8.6–25	203–466
R-MDA	16 (13–19)	22 (19–25)		273 (228–327)			
	7.7–41	8.0–24		120–725			
HMMA	162 (128–203)	2.8 (2.3–3.4)	18 (16–21)	2020 (1672–2441)	3559 (2929–4325)	70 (58–85)	1840 (1513–2239)
	60–539	1.5–8.0	7.2–35	908–5211	1450–8484	29–172	807–3958

*AUC* area under the plasma concentration-time curve, *AUC*_*∞*_ AUC from time zero to infinity, *AUC*_*24*_ from time 0 to 24, *CL/F* apparent total clearance, *C*_*max*_ maximum observed plasma concentration, *T*_*1/2*_ plasma half-life, *T*_*max*_ time to reach C_max_, *95%CI* 95% confidence interval, *V*_*z*_*/F* apparent volume of distribution.

### Correlations

Correlations between the drug plasma concentrations and subjective, cardiovascular, cortisol, and prolactin responses are shown in Supplementary Figs. S10–S13, respectively.

### Blinding

Participants could not distinguish effects of the active substances (Supplementary Table S9) after the treatment session or at the end-of-study visit. Placebo was correctly identified by 83% of participants after the study session.

## Discussion

The present controlled study was the first to directly compare acute effects of MDMA, *S*-, and *R*-MDMA. As hypothesized, *S*-MDMA induced greater subjective stimulation than *R*-MDMA. However, at the doses used *S*-MDMA also had greater effects than *R*-MDMA on many other mood scales. Contrary to our hypothesis, *R*-MDMA did not produce greater psychedelic effects than *S*-MDMA. We observed overall comparable effects of MDMA, *S*-MDMA, and *R*-MDMA with regard to effect strength and quality of the responses with minor differences. Specifically, *S*-MDMA induced overall slightly stronger effects and significantly greater bad drug effects, visual alterations, and synesthesia on the VAS, comparable psychedelic- and mystical-type alterations of mind on the 5D-ASC and MEQ, and comparable mood effects on the AMRS compared with MDMA. *S*-MDMA produced greater increases in blood pressure, cortisol, and prolactin compared with MDMA and was the only substance to significantly induce depressive symptoms 1–3 days after administration. The higher 250 mg *R*-MDMA dose produced lower subjective effects on most VASs, comparable psychedelic-like alterations on the 5D-ASC and MEQ, and more introversion on the AMRS compared with MDMA and *S*-MDMA.

Evidence from animal studies and human reports indicates that both enantiomers of MDMA are active and produce differential effects or are even reportedly needed to synergistically produce the full MDMA experience [13, 16, 17]. Based on animal data, we expected that *S*-MDMA and racemic MDMA would be overall equipotent in inducing stimulant-type and adverse effects in humans [9, 13, 16, 33] and thus selected the same dose of 125 mg *S*-MDMA and MDMA for the present comparison. However, other self-administration data in humans indicated that a 100 mg dose of *S*-MDMA induced similar “intoxication” to 125 mg racemic MDMA [21]. The present findings confirm a slightly higher potency of *S*-MDMA compared with MDMA and indicate that a 100 mg dose of *S*-MDMA would be equivalent to a 125 mg dose of racemic MDMA. Thus, the overall slightly greater subjective and cardiostimulant effects of *S*-MDMA in the present study may mainly reflect the 25% greater potency of *S*-MDMA compared with MDMA rather than any qualitative differences between *S*-MDMA and MDMA.

Nevertheless, supporting our primary hypothesis, *S*-MDMA exhibited more cardio- and psychostimulant effects than MDMA and *R*-MDMA in the present study, consistent with animal data [11]. The stronger increase in blood pressure in response to *S*-MDMA compared with *R*-MDMA may reflect the higher potency of *S*-MDMA to interact with the norepinephrine-transporter and release norepinephrine compared with *R*-MDMA [4, 34]. Additionally, *S*-MDMA was the only substance to significantly produce depressed mood ratings 1–3 days after drug administration, which could reflect greater transient serotonin depletion [35]. In the present study, we also observed significantly higher ratings of “drug high” after the administration of *S*-MDMA compared with *R*-MDMA. *S*-MDMA was found to be more potent than *R*-MDMA in maintaining self-administration in rhesus monkeys [17], and *S*-MDMA but not *R*-MDMA reinstated responding for amphetamine, indicative of greater abuse liability [12, 19]. *S*-MDMA may be more addictive in humans than *R*-MDMA, but we cannot exclude the possibility that the small differences between substances in the present study are dose-dependent rather than substance-dependent.

*R*-MDMA was expected to elicit more psychedelic-like effects compared with *S*-MDMA because of its higher potency to stimulate 5-HT_2A_ receptors [8]. However, in the present study, *R*-MDMA did not produce more psychedelic-like effects on the 5D-ASC or PES48/MEQ than *S*-MDMA or MDMA. Thus, we could not confirm our hypothesis that *R*-MDMA induces more psychedelic-like effects than *S*-MDMA at the doses used, although a higher dose of R-MDMA would need to be investigated. On the other hand, on the VAS, *S*-MDMA produced greater alterations of vision and greater audio-visual synesthesia than MDMA and *R*-MDMA, effects that would both be considered characteristic of psychedelics [36].

The therapeutic efficacy of MDMA might be enhanced by its ability to promote prosocial behaviors, foster openness, and facilitate a stronger therapeutic bond between the patient and therapist [2, 37, 38]. Animal studies found increases in social interaction in response to MDMA and higher doses of *R*-MDMA but only weak or no prosocial effects of *S*-MDMA [15, 39]. In the present first study in humans, all substances increased VAS ratings of “talkative,” “open,” “trust,” “I feel close to others,” and “I want to be with others” compared with placebo, but *S*-MDMA induced higher ratings on all these scales compared with *R*-MDMA at both doses. All substances produced comparable increases in ratings of feelings of “connectedness” on the PES48 compared with placebo. Thus, the present findings do not indicate greater prosocial effects of *R*-MDMA compared with MDMA or *S*-MDMA.

Oxytocin has overlapping social cognitive effects with MDMA [2, 40–42] and contributes to acute subjective effects of MDMA [1]. Cortisol and prolactin could be considered biomarkers of the serotonergic activity of MDMA [43]. In the present study, all substances increased circulating levels of oxytocin, cortisol, and prolactin. *S*-MDMA produced greater increases in oxytocin and cortisol compared with *R*-MDMA. *S*-MDMA also released prolactin at least as effectively as *R*-MDMA, in contrast to a study in rhesus monkeys [10]. The present findings align with stronger stimulation of the serotonin system by *S*-MDMA compared with *R*-MDMA at the doses used in the present study and are consistent with the greater serotonergic potency (but not selectivity) of *S*-MDMA compared with *R*-MDMA [4, 34].

Animal studies reported no hyperthermic effects of *R*-MDMA in mice or rats [14–16]. However, we found similar minimal increases in body temperature after *S*-MDMA and *R*-MDMA in the present human study.

Based on preliminary human data, the potency of *R*-MDMA was considered lower than MDMA and *S*-MDMA, with an effective dose “that might lie in the vicinity of 300 mg” [21]. Subjective effects of the *R*-MDMA doses that were used in the present study were lower than the 125 mg MDMA and 125 mg *S*-MDMA doses and indicate that a 300 mg dose may induce a comparable overall response to 125 mg MDMA or 100 mg *S*-MDMA. Thus, we would consider *S*-MDMA to be 1.25-fold more potent than MDMA and *R*-MDMA to be 2.4-fold less potent than MDMA. The in vitro potency of *S*-MDMA to release norepinephrine [34] or interact with the norepinephrine transporter was 4-fold higher compared with *R*-MDMA, predicting an approximately 4-fold higher potency in vivo [44].

Pharmacokinetics of *R*- and *S*-MDMA in humans have only been described after the administration of racemic MDMA [45–47]. After MDMA administration, *R*-MDMA had higher plasma concentrations (C_max_ and area under the curve) and an extended half-life compared with *S*-MDMA [45–47]. The present study confirmed the greater plasma exposure and longer elimination half-life of *R*-MDMA compared with *S*-MDMA after the administration of racemic MDMA. Additionally, the present study characterized pharmacokinetics of *S*-MDMA and *R*-MDMA in the absence of interactions with the other enantiomer. The elimination half-life of *S*-MDMA was 4.1 h when it was administered alone but 5.1 h when it was administered with *R*-MDMA in the form of racemic MDMA. The elimination half-life of *R*-MDMA was 12 and 14 h for the 125 and 250 mg doses of pure *R*-MDMA, respectively, indicating an increase with dose. Additionally, the formation of *R*-MDA from *R*-MDMA was dose-proportional, whereas the formation of HMMA from *R*-MDMA decreased with higher doses of *R*-MDMA. Although the dose of *R*-MDMA was doubled from 125 mg to 250 mg, the HMMA concentration did not double as well. Altogether, the data confirm that *R*-MDMA inhibits CYP2D6, thereby inhibiting its own inactivation to HMMA [48] similar to MDMA [49]. The present findings that the half-life of *S*-MDMA becomes shorter when it is administered without the *R*-enantiomer and that the HMMA concentrations were elevated when *S*-MDMA was administered compared with when *R*-MDMA was administered, indicating potentially less inhibition of CYP2D6 by *S*-MDMA.

We also showed that MDMA and MDA in humans did not undergo chiral inversion [32]. Thus, although HMMA was not enatioselectively measured, it can be assumed that only *S*- and *R*-HMMA are formed after *S*- and *R*-MDMA administration, respectively.

The present study has several strengths. A relatively large study sample (*n* = 24) and powerful within-subjects comparisons were used in a randomized double-blind design. Excellent blinding between *S*-MDMA, *R*-MDMA, and MDMA was confirmed. Two doses of the main substance of interest, *R*-MDMA, were included. We also included equal numbers of male and female participants. We used a wide range of internationally established psychometric outcome measures. Plasma concentrations were determined at close intervals in all participants and analyzed with validated achiral and chiral methods [32].

Notwithstanding its strengths, the present study also has limitations. To avoid too many exposures to MDMA, we had to limit the use of doses for each substance. We used only one dose of *S*-MDMA and only two doses of *R*-MDMA and failed to use exactly equivalent doses of the different substances. Doses of 100 mg *S*-MDMA and 300 mg *R*-MDMA would have been more equivalent. Consequently, we cannot confirm whether the observed differences between substances were attributable to the use of non-equivalent doses or qualitative properties of the substances. The study used a highly controlled hospital setting and included only healthy volunteers. People in different environments and patients with psychiatric disorders may respond differently to these substances. The outcome measures might not have been sufficiently sensitive to capture all aspects of the substance experience and very subtle differences between acute effects of MDMA and its enantiomers.

## Conclusion

In conclusion, the present study found that racemic MDMA, *S*-MDMA, and *R*-MDMA induced overall similar qualitative subjective and adverse effects when dosed equivalently. *S*-MDMA may have slightly greater stimulant-like properties than MDMA and *R*-MDMA. The results indicate dose-equivalence with regard to overall acute effects of 125 mg MDMA, 100 mg *S*-MDMA, and 300 mg *R*-MDMA. The pharmacokinetic findings indicate that *R*-MDMA dose-dependently inhibits CYP2D6 and thus its own inactivation and the inactivation of *S*-MDMA when administered as racemic MDMA. Overall, the present findings do not presently indicate relevant beneficial effects of *R*-MDMA or *S*-MDMA over MDMA in substance-assisted therapy in patients.

## Supplementary information

Supplement
