# Supplementary material for: Acute effects of R-MDMA, S-MDMA, and racemic MDMA in a randomized double-blind cross-over trial in healthy participants
Source: Neuropsychopharmacology. 2024 Aug 23;50(2):362–71. doi: 10.1038/s41386-024-01972-6 (PMC11631982; doi:10.1038/s41386-024-01972-6)
Supplement: Supplementary file 1 — Supplement [file 41386_2024_1972_MOESM1_ESM.pdf]

## Supplement

## Methods

### *Sample size calculation*

Power analysis was performed with PASS®, Hintze J. Kaysville, Utah, US. A difference of 15% in the primary measures was considered clinically meaningful. A sample size of 16 achieves 80% power to detect a difference of 15% between the null hypothesis mean of 100% and the alternative hypothesis mean of 85% with an estimated standard deviation of 20% and with a significance level (alpha) of 0.05 using a two-sided one-sample t-test. The study would have been adequately powered for the primary endpoint with a sample size of 16. A minimal sample size of 20 accounted for additional endpoints although these analyses remain more exploratory and/or confirmatory of the primary endpoint findings but using alternative and additional measures. Based on our experience with a similar study we assume a screening-failure rate of 25% and we expect to screen 36 subjects to include 24 in the study.

### *Prior and current substance use*

We aimed at including persons with no or limited previous drug experience as similar substance use experiences are mostly observed in patients treated with MDMA. Thus, persons with no prior experience were included as well as persons with a few prior experiences. However, we excluded persons with > 20 prior illicit substance uses. There was no restriction on prior use of $\Delta^9$-tetrahydrocannabinol (THC) as THC-use is prevalent. However, persons with any substance use disorder including THC were excluded.

Eleven participants had previously used MDMA (1–9 times), 11 participants had used a psychedelic (1–10 times), and nine participants had used a stimulant, including cocaine (eight participants, 1–5 times), amphetamine (six participants, 1–3 times), and methylphenidate (three participants, once). Five participants had used nitrous oxide (1–5 times), four participants had used ketamine (1–3 times), and one participant had used 4-bromo-2,5-dimethoxyphenethylamine (once). Six participants had never used any illicit drugs, with the exception of THC. One participant smoked two tobacco cigarettes daily and three participants smoked occasionally. Seven-teen participants drank alcohol. Mean+SD consumption of alcohol was 2.8+3.0 standard drinks per week (range: 1-10). Twenty-two participants had use cannabis (Table S1).

Table S1. Life-time prevalence of illicit drug use and current substance use

| Subject | MDMA | hallucinogens | sedatives | stimulants | opioids | THC | nicotine | alcohol | caffeine |
|---|---|---|---|---|---|---|---|---|---|
| 1 | 5 | 5 | 3 | 3 | 1 | ~200 | 0 | 3 | 2 |
| 2 | 1 | 0 | 0 | 0 | 0 | 0 | 0 | 3 | 4 |
| 3 | 1 | 4 | 0 | 3 | 0 | ~180 | 0 | 10 | 3 |
| 4 | 1 | 0 | 0 | 0 | 0 | ~100 | 0 | 8 | 1 |
| 5 | 0 | 2 | 0 | 1 | 0 | ~100 | 0 | 2 | 2 |
| 6 | 0 | 0 | 0 | 0 | 0 | ~100 | 0 | 0 | 0 |
| 7 | 3 | 0 | 2 | 8 | 0 | ~69 | ~60 | 8 | 1 |
| 8 | 5 | 1 | 3 | 7 | 0 | ~500 | 0 | 5 | 2 |
| 9 | 0 | 0 | 2 | 0 | 1 | 5 | 0 | 3 | 2 |
| 10 | 0 | 0 | 0 | 0 | 0 | 0 | 0 | 0 | 0 |
| 11 | 0 | 1 | 0 | 0 | 0 | ~100 | 0 | 0 | 2 |
| 12 | 0 | 0 | 5 | 1 | 0 | 20 | 0 | 0 | 1 |
| 13 | 0 | 0 | 0 | 0 | 0 | 20 | 1 – 5 | 3 | 1 |
| 14 | 3 | 10 | 0 | 3 | 1 | ~1000 | 0 | 0 | 1 |
| 15 | 0 | 0 | 2 | 0 | 0 | 1 | 0 | 0 | 0 |
| 16 | 0 | 0 | 0 | 0 | 0 | 4 | 0 | 0 | 3 |
| 17 | 0 | 0 | 0 | 0 | 0 | 6 | 0 | 0 | 1 |
| 18 | 3 | 10 | 0 | 0 | 0 | > 10.000 | 5 – 20 | 5 | 4 |
| 19 | 6 | 1 | 1 | 5 | 0 | ~1000 | ~80 | 5 | 10 |
| 20 | 9 | 9 | 1 | 0 | 0 | ~200 | 0 | 6 | 3 |
| 21 | 0 | 0 | 0 | 0 | 0 | 20 | 0 | 2 | 5 |
| 22 | 6 | 1 | 0 | 2 | 0 | ~50 | 0 | 2 | 0 |
| 23 | 0 | 7 | 0 | 0 | 0 | ~80 | 0 | 0 | 0 |
| 24 | 0 | 2 | 0 | 0 | 0 | 20 | 0 | 1 | 4 |

Values are times used in life, except nicotine (cigarettes per month), alcohol (units per week), and caffeine (cups per day)

*Subjective drug effects measurements*
*Visual Analog Scales (VASs)*

Subjective effects were assessed repeatedly using visual analog scales (VASs) [1,2]. 0.5 h before and 0, 0.25, 0.5, 0.75, 1, 1.5, 2, 2.5, 3, 3.5, 4, 5, 6, 7, 8, 9, and 24 h after drug administration. The VASs included “any drug effect”, “good drug effect”, “bad drug effect”, “stimulated”, “liking”, “feeling high”, “fear”, “alteration of vision”, “sounds seem to influence what I see”, “alteration of sense of time” and “ego dissolution” that were presented as 100-mm horizontal lines (0-100%), marked from “not at all” on the left to “extremely” on the right [1,3]. Further VASs included “happy”, “content”, “talkative”, “open”, “trust”, “I feel close to others” “I want to be alone”, and “I want to be with others”. These VASs were bidirectional and marked with “normal” in the middle at 0 mm and “not at all” (-50 mm) on the left and “extremely” (50 mm) on the right. The primary VAS outcome measure was “stimulation”. The VASs included in the present study have been repeatedly used and shown to be sensitive with MDMA [1,3-5]. Additionally the VAS “alteration of vision”, “sounds seem to influence what I see”, “alteration of sense of time” and “ego dissolution” were included because they were shown to be increased in response to the administration of different psychedelics [5-7] and to better capture potentially psychedelic-like effects of R-MDMA. The VAS can be completed relatively rapidly and easily by the participant even during the MDMA experience and allows for a valid prospective definition of the drug effects over time. They are sensitive and relatively simple measures. More complex assessments of the state of MDMA have to be performed primarily at the end of the session and include entire multi-item questionnaires. The VAS “any drug effect” is an overall effect measure to characterize the overall effect intensity and time course. The VAS “good drug effect” is an overall measure of effects subjectively considered positive and interrelated with other measures such as “drug liking”. The VAS “bad drug effect” is an overall measure of any negative effects and related to “fear”. The VAS “ego dissolution” was marked with the sentence: “the boundaries between myself and my surroundings seemed to blur”. This is also an item of the 5D-ASC (no. 71) which has been used as a simple measure of “ego dissolution” previously [8,9] and can be used repeatedly as a single VAS [1,10]. VASs were assessed each time MDMA blood concentrations were measured.

*Adjective Mood Rating Scale (AMRS)*

The Adjective Mood Rating Scale (AMRS) [11] was used 0.5 h before and 2.5, 5, and 9 h after drug administration. The AMRS is a validated 60-item Likert mood rating scale mainly use in Europe and consists of subscales including ratings on “well-being”, “anxiety”, “inactivity”, “extraversion”, “introversion”, and “emotional excitation”. It is suitable for repeated measurements of mood states. The short German EWL60S version was used [11]. The completion of the ratings under the effects of psychedelic substances is possible but difficult because it lasts several minutes. The scale was used in paper and pencil version, but it may be more suitable to use this measure verbally during states of markedly impaired concentration. The AMRS was included as a secondary measure because it could be considered a better validated measure of mood states and producing more defined ratings than the VAS and to support findings on the VAS (AMRS well-being considered similar to VAS good drug effects; AMRS anxiety considered similar to VAS fear).

*5 Dimension of Altered States of Consciousness (5D-ASC) scale*

The 5 Dimensions of Altered States of Consciousness (5D-ASC) scale [12,13] was used as the primary outcome measure for psychedelic-like effects and was administered 9 h after drug administration to retrospectively rate peak drug effects. The 5D-ASC scale measures altered states of consciousness and contains 94 items (visual analog scales). The instrument consists of five subscales/dimensions [12] and 11 lower-order scales [13]. The 5D-ASC dimension “Oceanic Boundlessness” (27 items) measures derealization and depersonalization associated with positive emotional states, ranging from heightened mood to euphoric

exaltation. The corresponding lower-order scales include "experience of unity," "spiritual experience," "blissful state," "insightfulness," and "disembodiment." The dimension "Anxious Ego Dissolution" (21 items) summarizes ego-disintegration and loss of self-control phenomena associated with anxiety. The corresponding lower-order scales include "impaired control of cognition" and "anxiety." The dimension "Visionary Restructuralization" (18 items) consists of the lower-order scales "complex imagery," "elementary imagery," "audio-visual synesthesia," and "changed meaning of percepts." Two additional dimensions describe "Auditory Alterations" (15 items) and "Reduction of Vigilance" (12 items). The total 3D-ASC score is the total of the three main dimensions "Oceanic Boundlessness", "Anxious Ego-Dissolution", and "Visionary Restructuralization" and can be used as a measure of the overall intensity of the alteration of the mind [9]. The scale is well-validated in German [12] and many other languages and widely used to characterize the subjective effects of various psychedelic drugs. In particular, the scale has been used by most research groups to psychometrically assess LSD, psilocybin and MDMA effects [1,2,7,14-18]. Furthermore, acute ratings on the 5D-ASC after administration of psilocybin and LSD have been used to predict long-term effects of psychedelic treatments in patients [19-21]. Ratings on the 5D-ASC have been shown to closely correlate with ratings on the Mystical Effects Questionnaire (MEQ, see below) [9] which is primarily used by research groups in the US [20].

*Psychedelic Experience Scale (PES) and Mystical Effects Questionnaire (MEQ)*

Mystical experiences were assessed 9 h after drug administration using the Psychedelic Experience Questionnaire/Scale (PES) [22] that represents a revalidation of the original 100-item States of Consciousness Questionnaire (SOCQ) [9,23] and includes the 43-item Mystical Effects Questionnaire (MEQ43) [23], the 30-item Mystical Effects Questionnaire (MEQ30) [24], and the 40-item Mystical Effects Questionnaire (MEQ40) [22]. The MEQ30 subscales are "mystical", "positive mood", "transcendence of time/space", and "ineffability" and their total provides the MEQ30 total score. Ten more items allow to derive the additional subscales "paradoxicality" and "connectedness" (40-item MEQ40). Eight more items allow to derive the additional "visual experience" and "distressing experience" subscales that together with all other subscales for the PES subscales (48 items from the 100-item SOCQ. Note that the full 100-item questionnaire was completed by the participants and only 48 items are needed to derive the validate subscales [22]. Future research could use the full 100-item scale (SOCQ) or just the 48-items needed for the PES analysis. The published German version was used [9,22]. The MEQ has been used in numerous experimental and therapeutic trials with psilocybin [20,23,25-31]. The MEQ has also been used in many experimental trials with LSD and MDMA [1,2,5,7,32,33] We derived the four scale scores of the newly validated revised 30-item MEQ: mystical, positive mood, transcendence of time and space, and ineffability [24].

## Results

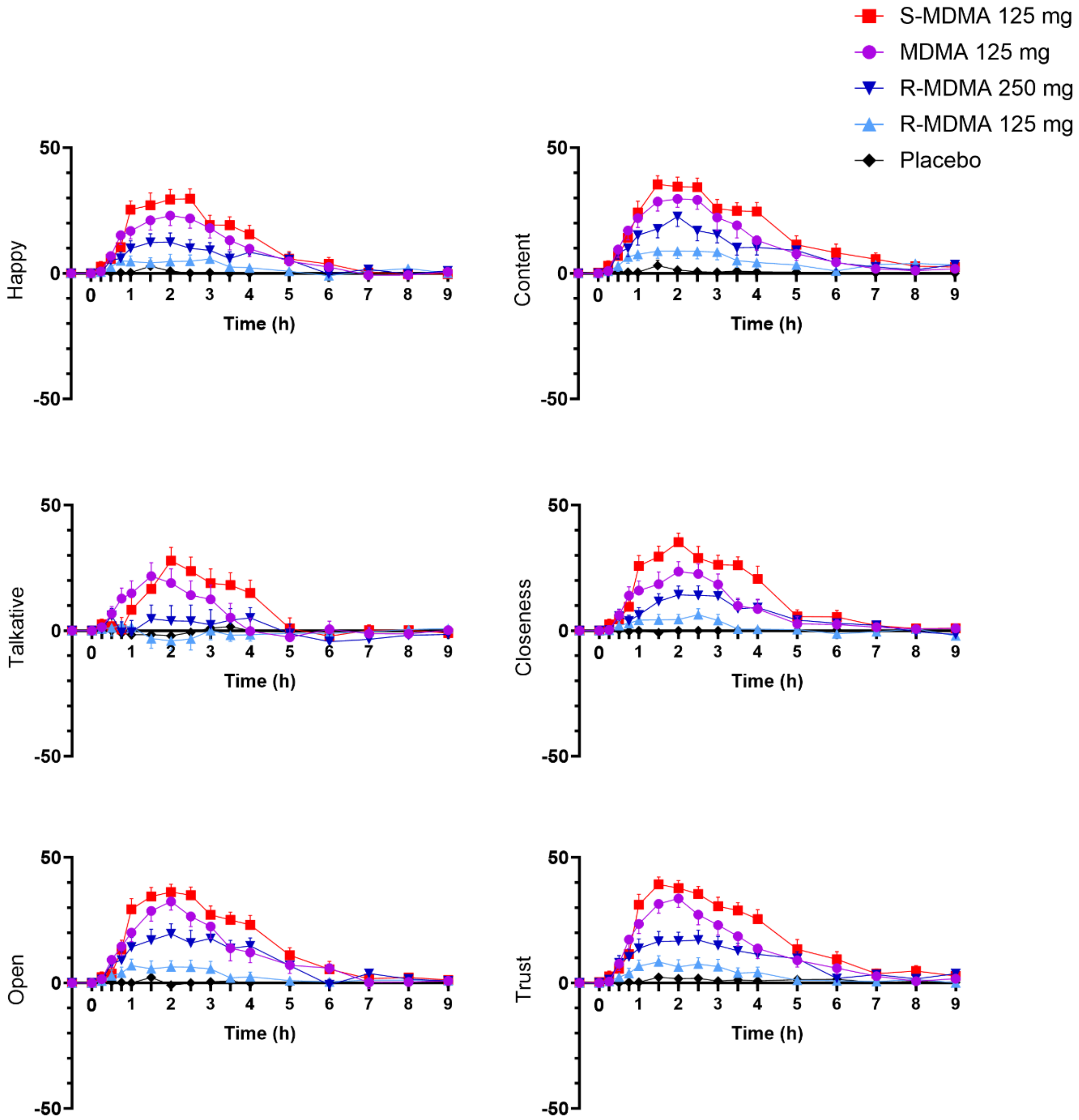


**Figure S1.** Acute subjective effects induced by 3,4-Methylendioxymethamphetamin (MDMA), *S*-MDMA, *R*-MDMA and placebo over time on the Visual Analog Scale (VAS). All substance conditions induced increases on the bidirectional VAS shown in this figure. *R*-MDMA at both doses induced weaker effects than *S*-MDMA and MDMA on all these VAS items. MDMA (125 mg), *S*-MDMA (125 mg), *R*-MDMA (125 mg), *R*-MDMA (250 mg) or placebo was administered at t = 0 h. The data are expressed as the mean ± SEM percentage of maximally possible scale scores in 24 subjects. The corresponding maximal responses and statistics are shown in Table 1.

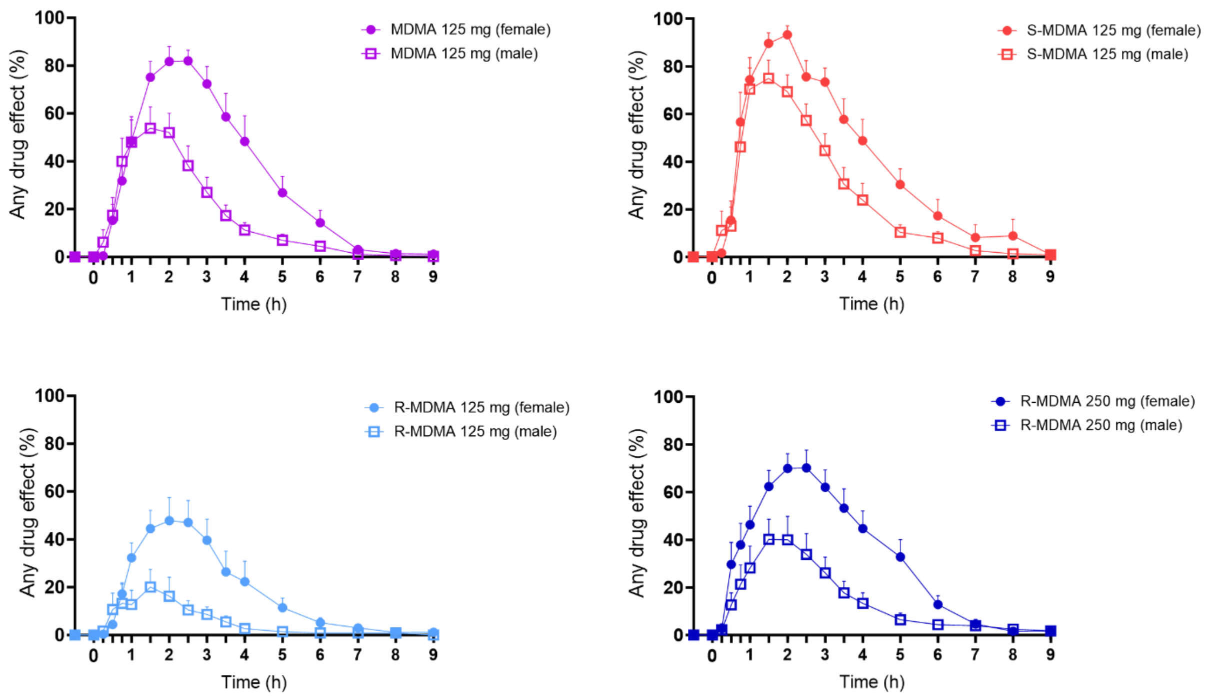


**Figure S2.** Sex differences in "any drug effects" of MDMA, *S*-MDMA, and *R*-MDMA on the VAS. Female participants reported stronger "any drug effects" than male participants across all substance conditions. However, when adjusting for weight, the difference in "any drug effects" between female and male became non-significant and is therefore driven by the lower body weight of women compared with men and the higher doses of the substances per kg body weight in women compared with men. The weight-dependent greater plasma levels were observed with all substances and indicate that lower doses could be used in humans with lower body weight. In the present study, the weight and sex-differences did not confound the results because treatments were compared within-subjects. MDMA (125 mg), *S*-MDMA (125 mg), *R*-MDMA (125 mg), *R*-MDMA (250 mg) or placebo was administered at t = 0 h. The data are expressed as the mean ± SEM percentage of maximally possible scale scores in 24 participants (12 female, 12 male). The corresponding maximal responses are shown in Table S2.

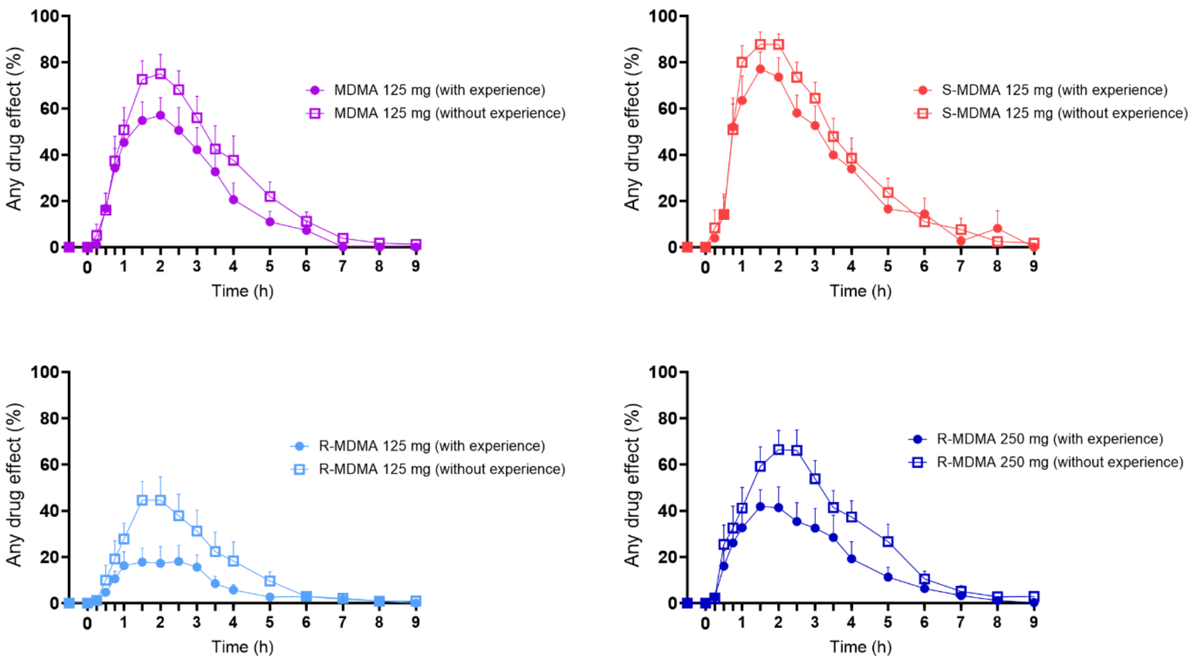


**Figure S3.** Differences in "any drug effects" of MDMA, *S*-MDMA, and *R*-MDMA between participants with and without previous MDMA experience on the VAS. Participants without prior MDMA experience indicated slightly higher "any drug effects" compared with participants with previous MDMA experience, although the differences were not statistically significant. MDMA (125 mg), *S*-MDMA (125 mg), *R*-MDMA (125 mg), *R*-MDMA (250 mg) or placebo was administered at t = 0 h. Prior drug experience did not confound the comparison between substances because there were no relevant order effects, and the order of the substance administration was balanced across the study. The data are expressed as the mean ± SEM percentage of maximally possible scale scores in 24 subjects, with 11 participants having prior MDMA experience and 13 participants having no previous experience with MDMA. The corresponding maximal responses are shown in Table S3.

We also compared the subjective effects in the 18 participants with prior illicit substance experience with those in 6 participants with no prior illicit substance experience (with the exception of THC). The findings were similar to those shown above in Figure S3 for the MDMA-experienced and MDMA-naïve participants with no statistical difference. Furthermore, there may be confounding by other differences besides prior drug experience such as sex.

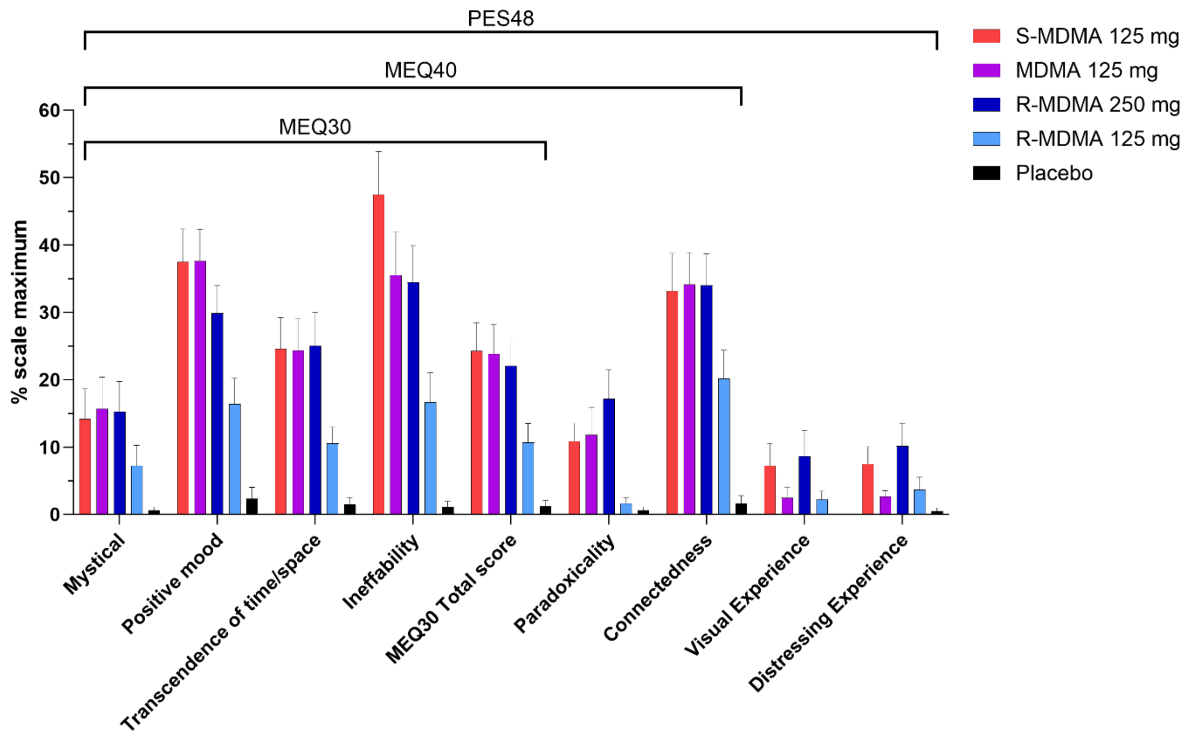


**Figure S4.** Acute mystical-type experiences on the Psychedelic Experience Scale (PES) and the 30- and 40-item Mystical Effects Questionnaire (MEQ30 and MEQ40, respectively). MDMA (125 mg), *S*-MDMA (125 mg) and *R*-MDMA (250 mg) induced overall comparable effects on the MEQ30, the MEQ40 and the 48-item PES48. 125 mg *R*-MDMA only induced effects on the subscales positive mood and ineffability on the MEQ30 and the ME30 total score. Additionally, 125 mg *R*-MDMA also induced effects on the connectedness subscale of the MEQ40 which were comparable to the other substances. The data are expressed as the mean ± SEM percentage of maximally possible scale scores in 24 subjects. Statistics are shown in Supplementary Table S6.

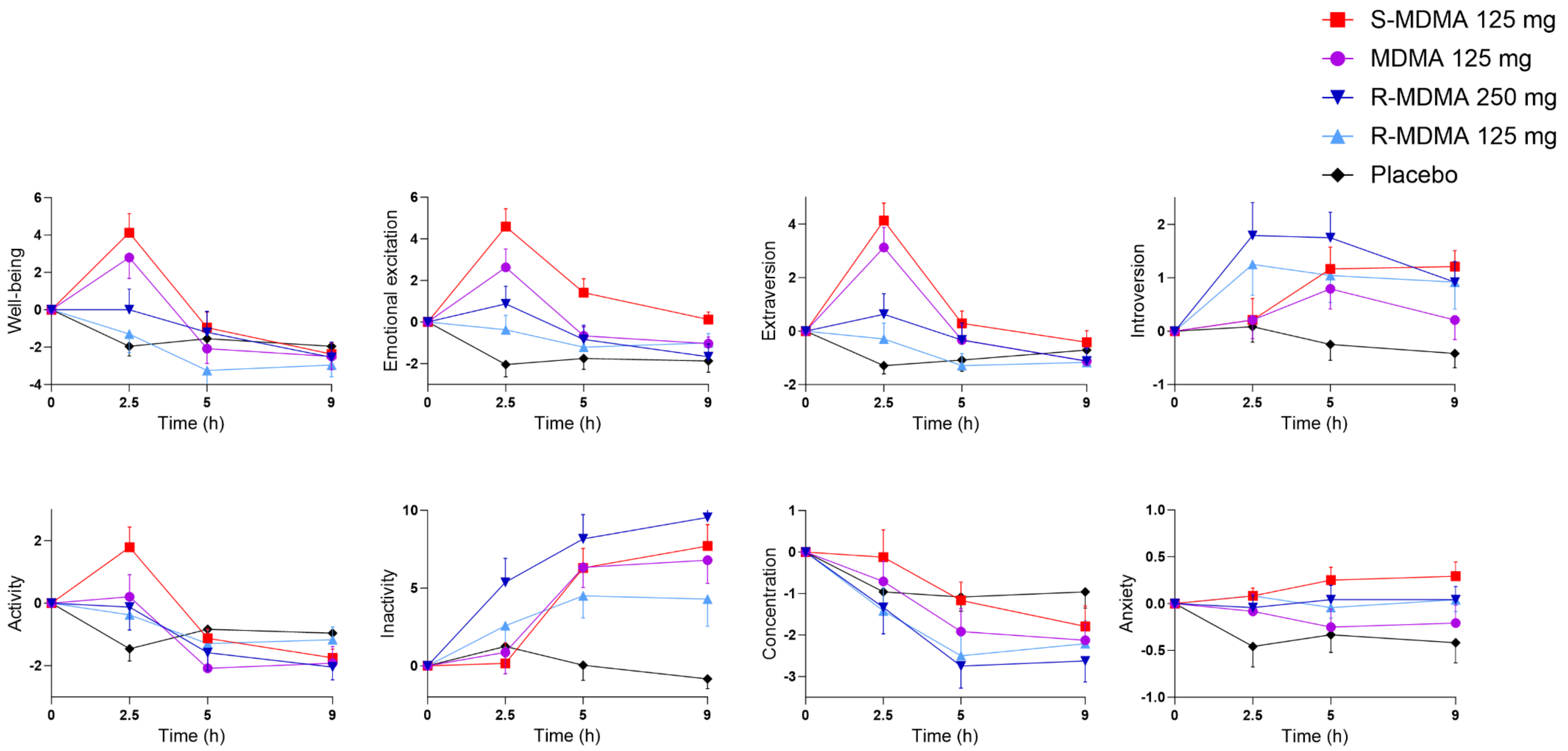


**Figure S5**. Subjective effects over time on the Adjective Mood Rating Scale (AMRS). MDMA and *S*-MDMA both increased well-being and emotional excitation and *S*-MDMA induced more emotional excitation than both doses of *R*-MDMA. MDMA, *S*-MDMA and 250 mg *R*-MDMA all significantly induced inactivity and extraversion. Introversion was induced by all enantiomer conditions but not with MDMA itself. 250 mg *R*-MDMA induced more "introversion" than MDMA. Only *S*-MDMA induced self-rated anxiety and none of the conditions changed values in activity and concentration. MDMA, *S*-MDMA, 125 mg *R*-MDMA, 250 mg *R*-MDMA or placebo was administered at t = 0 h. The data are expressed as mean ± SEM changes from baseline in 24 subjects. The corresponding maximal effects and statistics are shown in Supplementary Table S7.

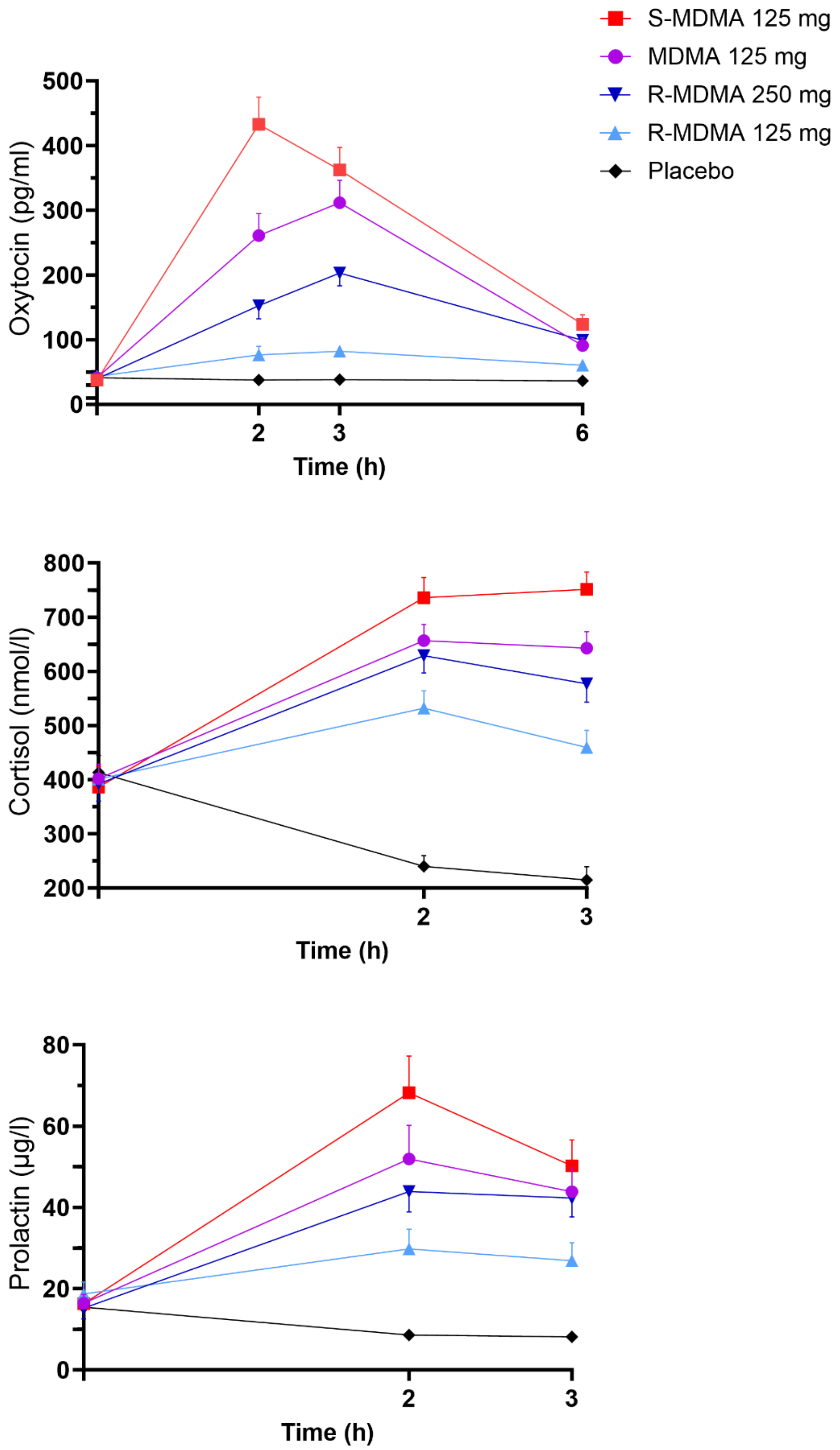


**Figure S6.** Plasma concentrations of oxytocin, cortisol, and prolactin with MDMA, *S*-MDMA. *R*-MDMA, and placebo. All substances increased oxytocin, cortisol, and prolactin compared with placebo. *S*-MDMA increased oxytocin and cortisol release more compared to MDMA and both doses of *R*-MDMA. Prolactin release was increased more with *S*-MDMA compared to MDMA and 125 mg *R*-MDMA. The data are expressed as mean ± SEM. 125 mg MDMA, 125 mg *S*-MDMA, 125 mg *R*-MDMA, 250 mg *R*-MDMA or placebo was administered at t = 0 h. The corresponding maximal effects and statistics are shown in Table 1.

a

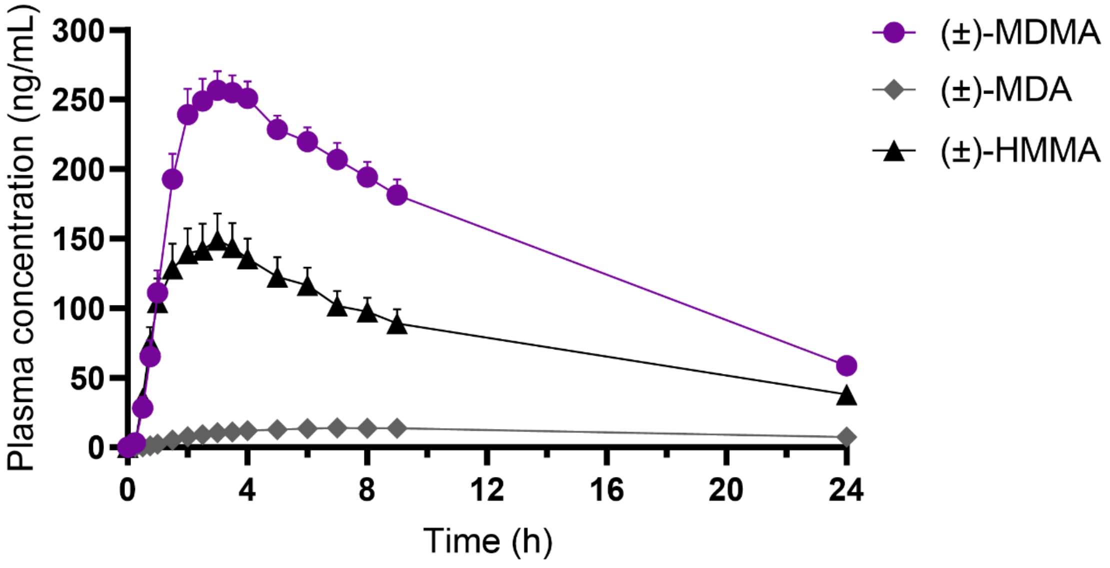


b

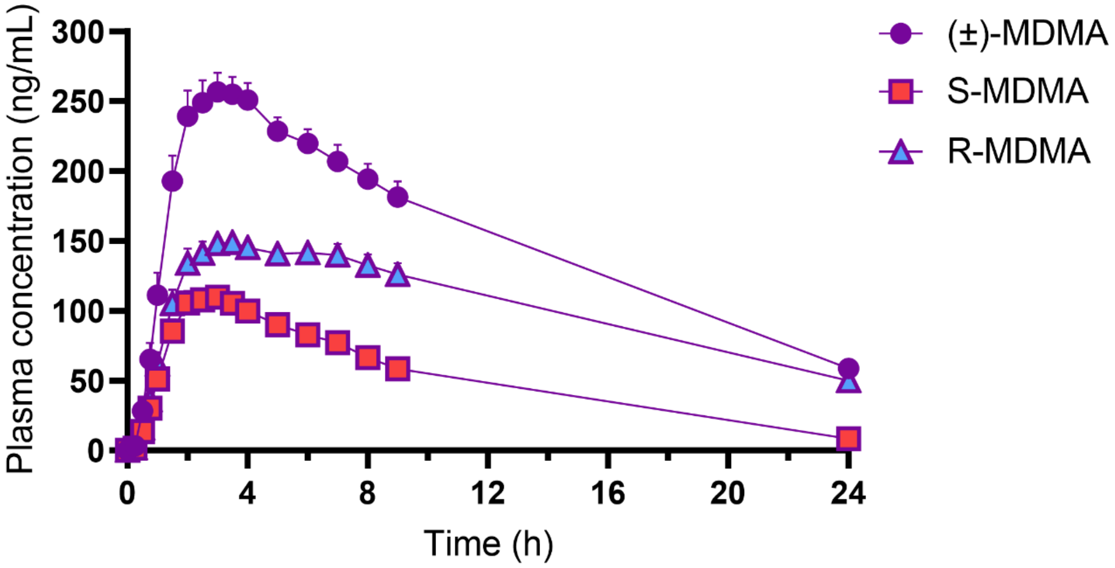


**Figure S7.** (**a**) Plasma concentrations of (±)-MDMA and its metabolites (±)-MDA and (±)-HMMA when 125 mg (±)-MDMA was administered. HMMA concentrations were determined after enzymatic deglucuronidation. (**b**) Plasma concentrations of the racemic (±)-MDMA and its enantiomers *S*- and *R*-MDMA were measured separately after the administration of 125 mg (±)-MDMA. Plasma concentration ($C_{max}$ and area under the curve (AUC)) was higher, and half-life ($t_{1/2}$) was longer for the *R*-enantiomer compared to the *S*-enantiomer. The data are expressed as mean ± SEM. (±)-MDMA was administered at t = 0 h. The corresponding pharmacokinetic parameters were determined by non-compartmental analysis and are shown in Table 2.

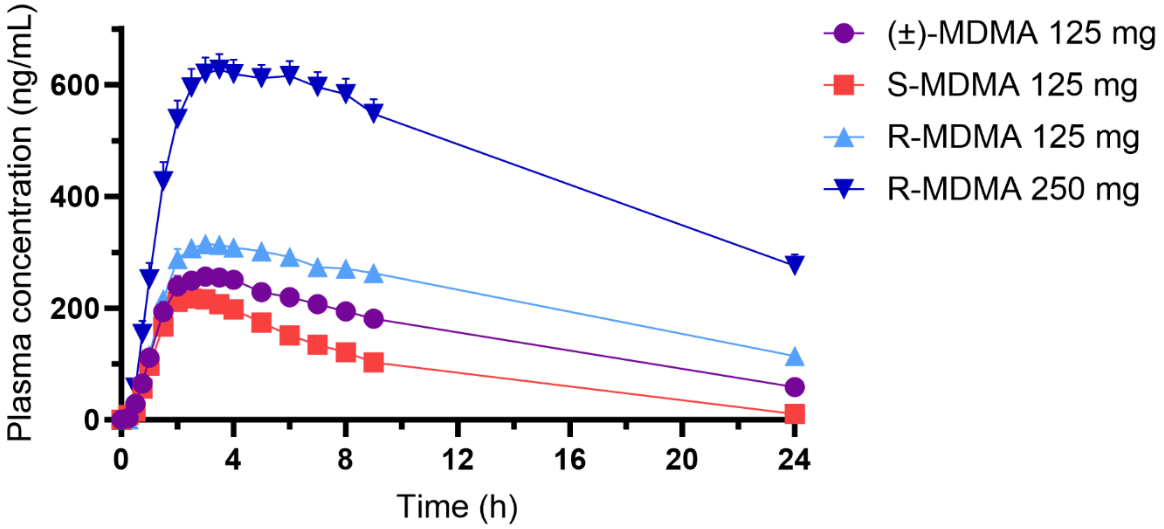


**Figure S8.** Plasma concentrations of (±)-MDMA, *S*-MDMA and *R*-MDMA after administration of the respective substance. The enantiomer *R*-MDMA reaches higher plasma concentrations and *S*-MDMA lower plasma concentrations compared with (±)-MDMA when administered at the same dose. The data are expressed as mean ± SEM. (±)-MDMA (125 mg), *S*-MDMA (125 mg), *R*-MDMA (125 mg), or *R*-MDMA (250 mg) was administered at t = 0 h. The corresponding pharmacokinetic parameters were determined by non-compartmental analysis and are shown in Table 2.

**a**

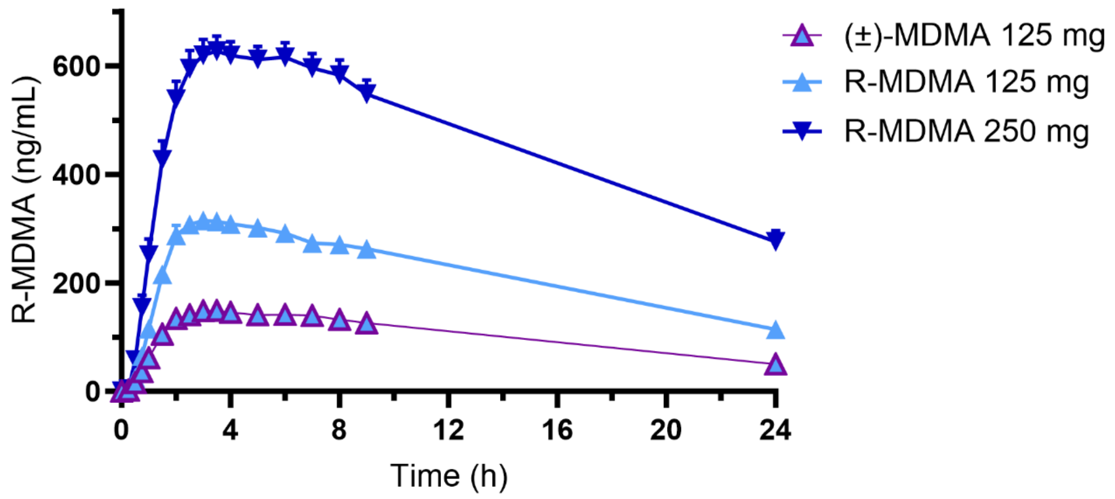


**b**

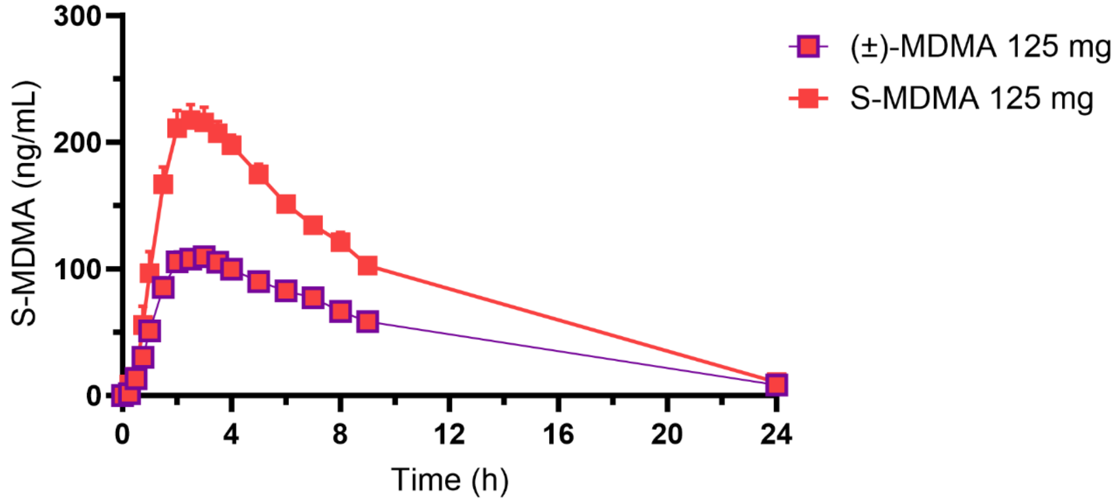


**Figure S9.** (**a**) Plasma concentration of *R*-MDMA when 125 mg (±)-MDMA, 125 mg *R*-MDMA and 250 mg *R*-MDMA was administered. The half-life of *R*-MDMA increases with higher doses of *R*-MDMA given. (**b**) Plasma concentration of *S*-MDMA when 125 mg (±)-MDMA and 125 mg *S*-MDMA were administered. Administration of only the *S*-enantiomer shortened the half-life by one hour compared to the administration as racemic (±)-MDMA. The data are expressed as mean ± SEM. (±)-MDMA (125 mg), *S*-MDMA (125 mg), *R*-MDMA (125 mg), or *R*-MDMA (250 mg) was administered at t = 0 h. The corresponding pharmacokinetic parameters were determined by non-compartmental analysis and are shown in Table 2.

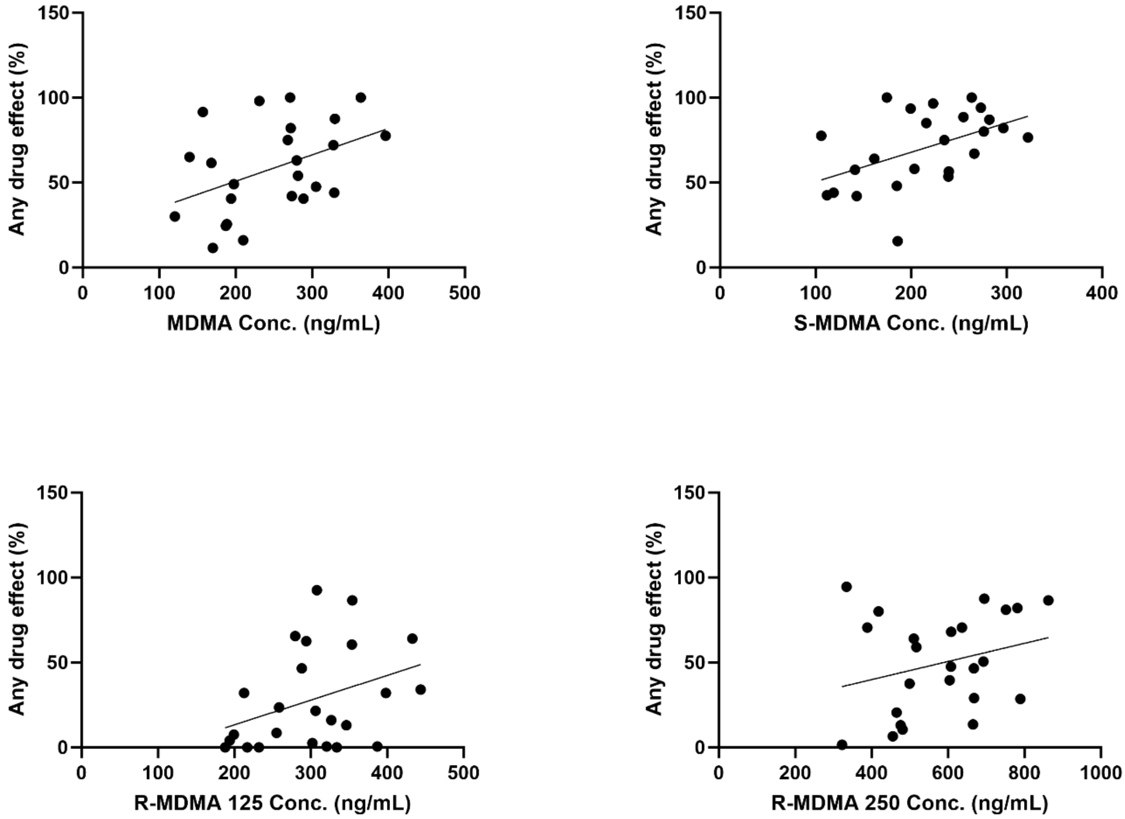


**Figure S10.** Correlations between substance plasma concentrations and the subjective “Any drug effect” rating. The data points represent the “any drug effect” on the VAS at time points 2 and 3 hours (expressed as the mean) as a measure of the subjective peak response and at the same time points where the cortisol and prolactin concentrations were measured, as well as the mean plasma substance concentrations at these time points. Pearson correlation analyses with a significance criterion of $p<0.05$ were conducted. Correlation coefficients and p-values for the substances were: MDMA (r=0.43, $p=0.04$), *S*-MDMA (r=0.48, $p=0.02$), R-MDMA 125 mg (r=0.36, $p=0.08$), and R-MDMA 250 mg (r=0.27, $r=0.2$).

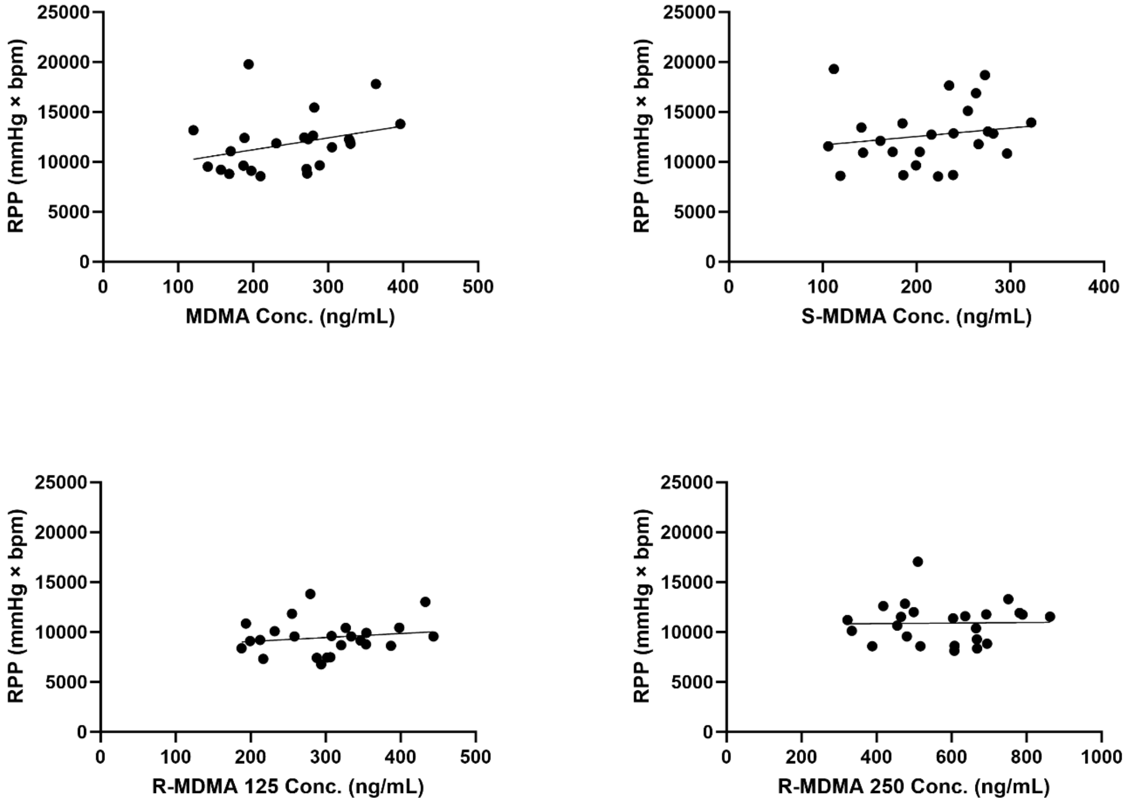


**Figure S11.** Correlations between substance plasma concentration and the cardiovacular response expressed by the rate pressure product (RPP). The data points represent the rate pressure product at time points 2 and 3 hours (expressed as the mean) as a measure of the autonomic peak response and at the same time points where the cortisol and prolactin concentrations were measured, as well as the mean plasma substance concentrations at these time points. Pearson correlation analyses with a significance criterion of *p<0.05* were conducted. Correlation coefficients and p-values for the substances were: MDMA (r=0.31, *p=0.14*), *S*-MDMA (r=0.17, *p=0.43*), R-MDMA 125 mg (r=0.17, *p=0.43*), and R-MDMA 250 mg (r=0.02, *r=0.93*).

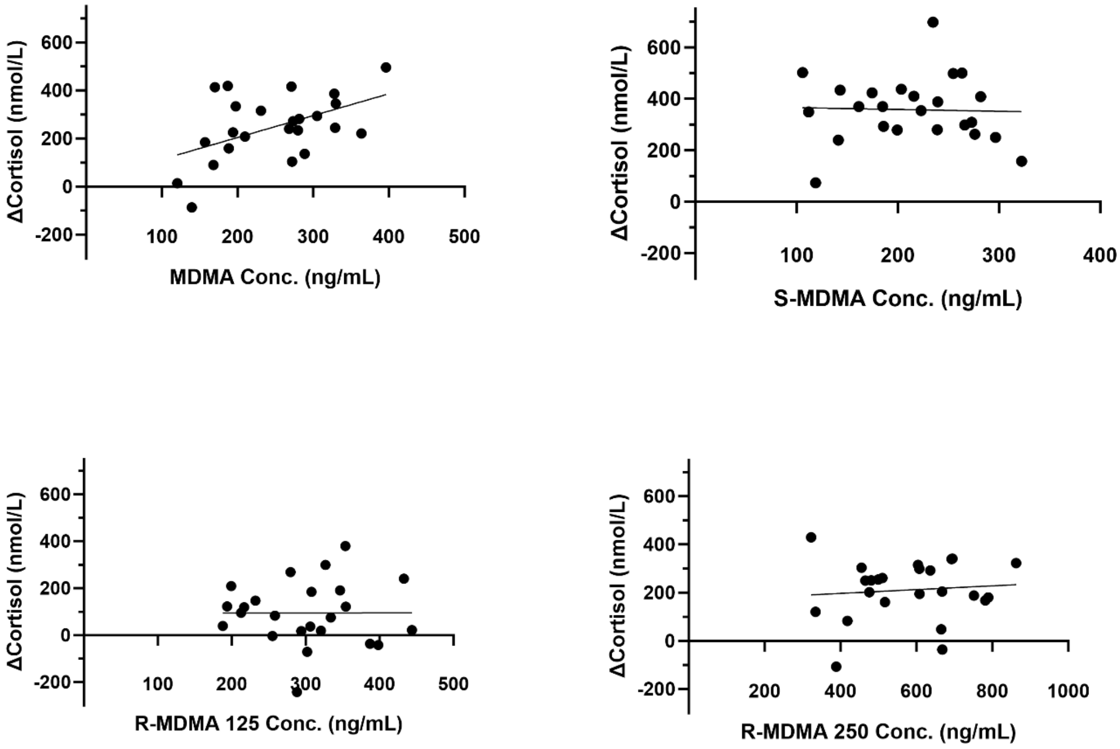


**Figure S12.** Correlations substance and cortisol plasma concentrations. The data points represent the change of cortisol levels from baseline to after 2 and 3 hours (expressed as the mean), as well as the mean plasma substance concentrations at these time points. Pearson correlation analyses with a significance criterion of *p<0.05* were conducted. Correlation coefficients and p-values for the substances were: MDMA (r=0.50, *p=0.01*), *S*-MDMA (r=-0.03, *p=0.88*), *R*-MDMA 125 mg (r=0.001, *p=1.0*), and *R*-MDMA 250 mg (r=0.09, *p=0.66*), respectively.

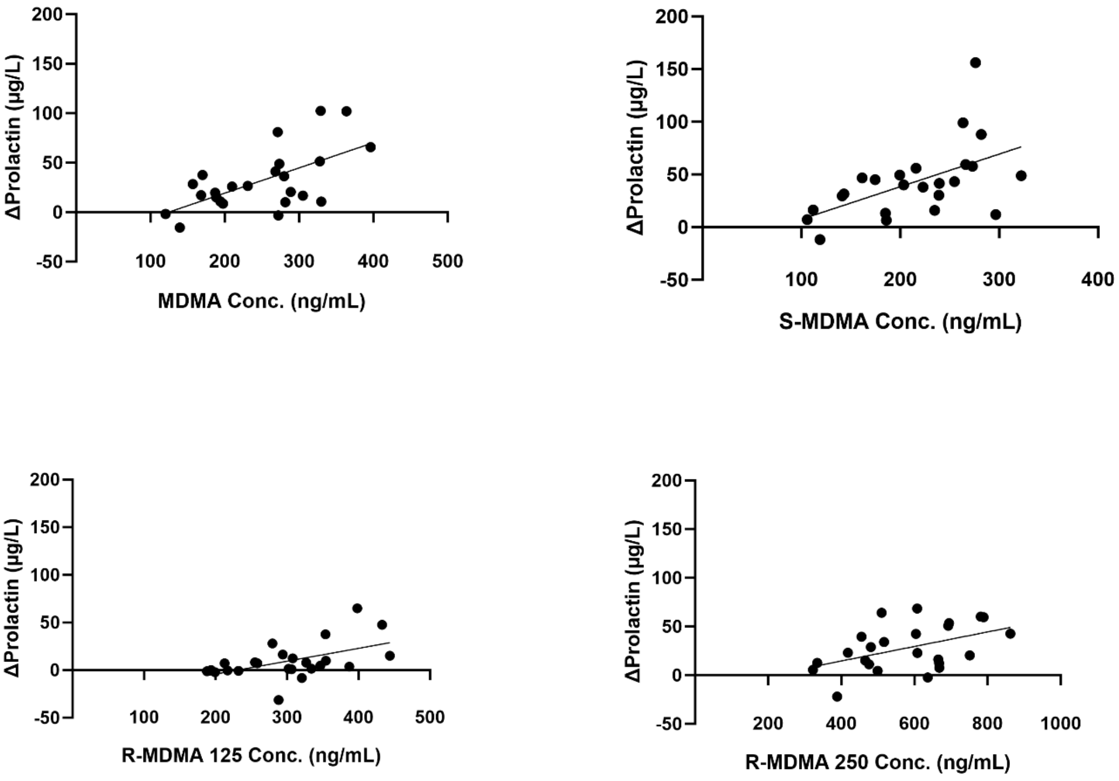


**Figure S13.** Correlations between substance and prolactin plasma concentrations. The data points represent the change of prolactin levels from baseline to after 2 and 3 hours (expressed as the mean), as well as the mean plasma substance concentrations at these time points. Pearson correlation analyses with a significance criterion of *p<0.05* were conducted. Correlation coefficients and p-values for the substances were: MDMA (r=0.62, *p=0.001*), *S*-MDMA (r=0.54, *p=0.01*), *R*-MDMA 125 mg (r=0.51, *p=0.01*), and *R*-MDMA 250 mg (r=0.47, *p=0.02*), respectively.

**Table S2. Sex differences in mean acute subjective effects of MDMA, S-MDMA, R-MDMA and placebo on the Visual Analogue Scale (VAS)**

| | | Placebo (mean ± SEM) | 125 mg R-MDMA (mean ± SEM) | 250 mg R-MDMA (mean ± SEM) | 125 mg MDMA (mean ± SEM) | 125 mg S-MDMA (mean ± SEM) |
|---|---|---|---|---|---|---|
| Visual Analog Scale (VAS, %max) | | | | | | |
| Unidirectional Scales (0-100) | | | | | | |
| **Any drug effect** | $\Delta E_{max}$ | 4.8 ± 2.1 | 42 ± 7.3 | 66 ± 6.1 | 77 ± 5.3 | 90 ± 3.1 |
| female | $\Delta E_{max}$ | 6.3 ± 4.1 | 57 ± 9.0 | 83 ± 4.5 | 91 ± 3.8 | 97 ± 2.3 |
| male | $\Delta E_{max}$ | 3.3 ± 1.1 | 27 ± 10 | 50 ± 9.2 | 63 ± 8.1 | 82 ± 5.0 |
| **Good drug effect** | $\Delta E_{max}$ | 7.9 ± 4.1 | 43 ± 7.1 | 68 ± 6.6 | 78 ± 5.1 | 90 ± 3.8 |
| female | $\Delta E_{max}$ | 7.7 ± 6.0 | 54 ± 10 | 82 ± 7.4 | 87 ± 5.6 | 93 ± 5.2 |
| male | $\Delta E_{max}$ | 8.1 ± 5.8 | 32 ± 9.4 | 55 ± 9.8 | 69 ± 8.0 | 87 ± 5.6 |
| **Bad drug effect** | $\Delta E_{max}$ | 0.5 ± 0.3 | 14 ± 5.1 | 19 ± 4.6 | 20 ± 5.9 | 39 ± 7.0 |
| female | $\Delta E_{max}$ | 0.8 ± 0.6 | 21 ± 7.5 | 29 ± 6.5 | 30 ± 8.0 | 40 ± 8.3 |
| male | $\Delta E_{max}$ | 0.3 ± 0.2 | 8.1 ± 6.7 | 9.9 ± 5.3 | 11 ± 8.2 | 39 ± 12 |
| **I like the effect** | $\Delta E_{max}$ | 7.7 ± 3.8 | 47 ± 7.1 | 68 ± 6.6 | 81 ± 5.0 | 91 ± 3.6 |
| female | $\Delta E_{max}$ | 8.1 ± 5.4 | 58 ± 9.6 | 86 ± 6.5 | 89 ± 5.5 | 94 ± 3.8 |
| male | $\Delta E_{max}$ | 7.3 ± 5.6 | 37 ± 9.7 | 50 ± 9.2 | 72 ± 7.8 | 88 ± 6.0 |
| **Stimulated** | $\Delta E_{max}$ | 2.5 ± 1.1 | 31 ± 6.9 | 60 ± 6.7 | 70 ± 6.7 | 88 ± 3.7 |
| female | $\Delta E_{max}$ | 1.8 ± 1.1 | 40 ± 10 | 74 ± 8.3 | 80 ± 6.7 | 91 ± 4.5 |
| male | $\Delta E_{max}$ | 3.3 ± 2.0 | 22 ± 9.1 | 46 ± 9.2 | 61 ± 11 | 84 ± 6.0 |
| **Drug high** | $\Delta E_{max}$ | 1.4 ± 0.6 | 29 ± 7.0 | 48 ± 7.6 | 73 ± 6.7 | 84 ± 5.1 |
| female | $\Delta E_{max}$ | 1.6 ± 1.0 | 37 ± 9.9 | 65 ± 9.7 | 85 ± 6.9 | 93 ± 4.5 |
| male | $\Delta E_{max}$ | 1.3 ± 0.9 | 20 ± 9.7 | 31 ± 9.7 | 61 ± 11 | 75 ± 8.7 |
| **Fear** | $\Delta E_{max}$ | 0.0 ± 0.0 | 4.9 ± 4.2 | 5.8 ± 3.4 | 5.9 ± 3.4 | 19 ± 6.9 |
| female | $\Delta E_{max}$ | 0.0 ± 0.0 | 9.0 ± 8.3 | 4.8 ± 2.8 | 6.2 ± 6.0 | 21 ± 11 |
| male | $\Delta E_{max}$ | 0.1 ± 0.1 | 0.8 ± 0.7 | 6.8 ± 6.3 | 5.6 ± 3.6 | 16 ± 8.6 |
| **Alteration of vision** | $\Delta E_{max}$ | 3.0 ± 1.6 | 24 ± 6.9 | 37 ± 6.7 | 54 ± 7.8 | 74 ± 6.7 |
| female | $\Delta E_{max}$ | 4.4 ± 3.1 | 28 ± 10 | 47 ± 9.5 | 60 ± 11 | 79 ± 9.1 |
| male | $\Delta E_{max}$ | 1.7 ± 1.0 | 20 ± 9.6 | 27 ± 8.8 | 48 ± 11 | 70 ± 10 |
| **Alteration of sense of time** | $\Delta E_{max}$ | 2.2 ± 2.0 | 28 ± 7.2 | 46 ± 7.7 | 60 ± 6.8 | 73 ± 6.6 |
| female | $\Delta E_{max}$ | 4.0 ± 4.0 | 37 ± 10 | 61 ± 10 | 73 ± 7.7 | 87 ± 7.5 |
| male | $\Delta E_{max}$ | 0.4 ± 0.3 | 19 ± 9.9 | 32 ± 10 | 47 ± 10 | 58 ± 9.5 |
| **Audio-visual synesthesia** | $\Delta E_{max}$ | 4.6 ± 4.2 | 15 ± 5.9 | 31 ± 6.8 | 29 ± 6.8 | 53 ± 8.3 |
| female | $\Delta E_{max}$ | 0.7 ± 0.7 | 17 ± 8.8 | 38 ± 9.2 | 30 ± 8.7 | 57 ± 12 |
| male | $\Delta E_{max}$ | 8.5 ± 8.3 | 13 ± 8.2 | 23 ± 10 | 29 ± 11 | 49 ± 12 |
| **Ego dissolution** | $\Delta E_{max}$ | 1.5 ± 0.8 | 22 ± 7.4 | 32 ± 7.4 | 41 ± 7.7 | 58 ± 8.2 |
| female | $\Delta E_{max}$ | 2.0 ± 1.4 | 28 ± 11 | 43 ± 10 | 48 ± 11 | 72 ± 11 |
| male | $\Delta E_{max}$ | 1.0 ± 1.0 | 16 ± 9.7 | 21 ± 10 | 33 ± 10 | 45 ± 12 |

$\Delta E_{max}$, maximal effect difference from baseline; N=24 (12 female, 12 male)

**Table S3. Differences in mean acute subjective effects of MDMA, S-MDMA, R-MDMA and placebo between participants with and without previous MDMA experience on the Visual Analogue Scale (VAS)**

| | | Placebo | 125 mg R-MDMA | 250 mg R-MDMA | 125 mg MDMA | 125 mg S-MDMA |
|---|---|---|---|---|---|---|
| | | (mean ± SEM) | (mean ± SEM) | (mean ± SEM) | (mean ± SEM) | (mean ± SEM) |
| Visual Analog Scale (VAS, %max) | | | | | | |
| Unidirectional Scales (0-100) | | | | | | |
| **Any drug effect** | $\Delta E_{max}$ | 4.8 ± 2.1 | 42 ± 7.3 | 66 ± 6.1 | 77 ± 5.3 | 90 ± 3.1 |
| with experience | $\Delta E_{max}$ | 3.6 ± 1.2 | 25 ± 7.7 | 54 ± 9.1 | 68 ± 7.2 | 88 ± 5.1 |
| without experience | $\Delta E_{max}$ | 6.0 ± 3.8 | 57 ± 10 | 77 ± 7.3 | 84 ± 7.2 | 91 ± 3.9 |
| **Good drug effect** | $\Delta E_{max}$ | 7.9 ± 4.1 | 43 ± 7.1 | 68 ± 6.6 | 78 ± 5.1 | 90 ± 3.8 |
| with experience | $\Delta E_{max}$ | 2.2 ± 1.1 | 30 ± 8.2 | 54 ± 11 | 73 ± 7.2 | 87 ± 6.4 |
| without experience | $\Delta E_{max}$ | 13 ± 7.4 | 54 ± 10 | 80 ± 6.4 | 82 ± 7.3 | 92 ± 4.6 |
| **Bad drug effect** | $\Delta E_{max}$ | 0.5 ± 0.3 | 14 ± 5.1 | 19 ± 4.6 | 20 ± 5.9 | 39 ± 7.0 |
| with experience | $\Delta E_{max}$ | 0.9 ± 0.7 | 5.8 ± 3.6 | 14 ± 6.1 | 16 ± 6.4 | 37 ± 8.9 |
| without experience | $\Delta E_{max}$ | 0.2 ± 0.2 | 22 ± 8.6 | 24 ± 6.6 | 24 ± 9.6 | 42 ± 11 |
| **I like the effect** | $\Delta E_{max}$ | 7.7 ± 3.8 | 47 ± 7.1 | 68 ± 6.6 | 81 ± 5.0 | 91 ± 3.6 |
| with experience | $\Delta E_{max}$ | 3.0 ± 1.5 | 35 ± 9.0 | 55 ± 10 | 75 ± 7.4 | 89 ± 5.6 |
| without experience | $\Delta E_{max}$ | 12 ± 6.8 | 57 ± 10 | 79 ± 7.6 | 85 ± 6.8 | 93 ± 4.7 |
| **Stimulated** | $\Delta E_{max}$ | 2.5 ± 1.1 | 31 ± 6.9 | 60 ± 6.7 | 70 ± 6.7 | 88 ± 3.7 |
| with experience | $\Delta E_{max}$ | 1.0 ± 0.8 | 18 ± 5.7 | 51 ± 11 | 61 ± 10 | 85 ± 7.1 |
| without experience | $\Delta E_{max}$ | 3.8 ± 1.9 | 42 ± 11 | 68 ± 7.9 | 79 ± 8.6 | 90 ± 3.4 |
| **Drug high** | $\Delta E_{max}$ | 1.4 ± 0.6 | 29 ± 7.0 | 48 ± 7.6 | 73 ± 6.7 | 84 ± 5.1 |
| with experience | $\Delta E_{max}$ | 0.3 ± 0.2 | 12 ± 5.0 | 40 ± 12 | 63 ± 10 | 78 ± 9.5 |
| without experience | $\Delta E_{max}$ | 2.4 ± 1.1 | 43 ± 11 | 55 ± 9.8 | 82 ± 8.6 | 89 ± 5.0 |
| **Fear** | $\Delta E_{max}$ | 0.0 ± 0.0 | 4.9 ± 4.2 | 5.8 ± 3.4 | 5.9 ± 3.4 | 19 ± 6.9 |
| with experience | $\Delta E_{max}$ | 0.0 ± 0.0 | 0.7 ± 0.7 | 1.7 ± 1.7 | 0.6 ± 0.5 | 22 ± 12 |
| without experience | $\Delta E_{max}$ | 0.1 ± 0.1 | 8.4 ± 7.7 | 9.2 ± 6.0 | 10 ± 6.1 | 16 ± 8.1 |
| **Alteration of vision** | $\Delta E_{max}$ | 3.0 ± 1.6 | 24 ± 6.9 | 37 ± 6.7 | 54 ± 7.8 | 74 ± 6.7 |
| with experience | $\Delta E_{max}$ | 1.4 ± 0.7 | 8.5 ± 3.8 | 28 ± 9.3 | 43 ± 11 | 63 ± 12 |
| without experience | $\Delta E_{max}$ | 4.5 ± 2.9 | 37 ± 11 | 45 ± 9.2 | 63 ± 11 | 84 ± 5.5 |
| **Alteration of sense of time** | $\Delta E_{max}$ | 2.2 ± 2.0 | 28 ± 7.2 | 46 ± 7.7 | 60 ± 6.8 | 73 ± 6.6 |
| with experience | $\Delta E_{max}$ | 0.0 ± 0.0 | 13 ± 7.3 | 26 ± 9.3 | 48 ± 11 | 62 ± 11 |
| without experience | $\Delta E_{max}$ | 4.1 ± 3.7 | 41 ± 11 | 63 ± 9.9 | 70 ± 8.2 | 81 ± 7.2 |
| **Audio-visual synesthesia** | $\Delta E_{max}$ | 4.6 ± 4.2 | 15 ± 5.9 | 31 ± 6.8 | 29 ± 6.8 | 53 ± 8.3 |
| with experience | $\Delta E_{max}$ | 0.0 ± 0.0 | 4.5 ± 2.9 | 14 ± 4.9 | 20 ± 7.6 | 42 ± 11 |
| without experience | $\Delta E_{max}$ | 8.5 ± 7.7 | 23 ± 10 | 45 ± 11 | 37 ± 10 | 62 ± 12 |
| **Ego dissolution** | $\Delta E_{max}$ | 1.5 ± 0.8 | 22 ± 7.4 | 32 ± 7.4 | 41 ± 7.7 | 58 ± 8.2 |
| with experience | $\Delta E_{max}$ | 0.7 ± 0.5 | 11 ± 8.2 | 21 ± 10 | 30 ± 9.9 | 50 ± 13 |
| without experience | $\Delta E_{max}$ | 2.2 ± 1.5 | 31 ± 11 | 40 ± 11 | 50 ± 11 | 66 ± 10 |

$\Delta E_{max}$, maximal effect difference from baseline; with experience, with previous MDMA experience before the study; without experience, without previous MDMA experience before the study; N=24, $N_{with\ experience}$ = 11, $N_{without\ experience}$ = 13

**Table S4. Parameters characterizing the subjective drug effect-time curves of MDMA, S-MDMA and R-MDMA**

| | R-MDMA 125 mg | R-MDMA 250 mg | MDMA 125 mg | S-MDMA 125 mg |
|---|---|---|---|---|
| Time to onset (h) | 0.8 ± 0.1[α] (0.3 - 2.0) | 0.6 ± 0.1[γ] (0.1 - 1.8) | 0.6 ± 0.1 (0.04 - 1.1) | 0.5 ± 0.01 (0.03 - 1.0) |
| Time to offset (h) | 3.9 ± 0.5[α] (1.1 - 8.5) | 5.8 ± 0.8[γ] (1.7 - 22) | 4.8 ± 0.3 (2.3 - 7.8) | 5.2 ± 0.3 (2.4 - 9.0) |
| Time to maximal effect (h) | 1.7 ± 0.1[β] (0.5 - 3.5) | 1.8 ± 0.2 (0.8 - 5.0) | 1.9 ± 0.2 (0.8 - 4.0) | 1.4 ± 0.2 (0.3 - 4.0) |
| Effect duration (h) | 3.5 ± 0.5[α] (0.7 - 8.0) | 5.2 ± 0.8[γ] (1.0 - 21) | 4.2 ± 0.3 (1.5 - 7.0) | 4.7 ± 0.3 (1.8 - 8.5) |
| Maximal effect (%) | 46 ± 7.4[β] (3 - 100) | 66 ± 6.1 (4 - 100) | 77 ± 5.3 (22 - 100) | 90 ± 3.1 (51 - 100) |
| AUEC (h*pg/mL) | 115 ± 22[β] (1.9 - 336) | 216 ± 28 (6.2 - 553) | 225 ± 24 (37 - 455) | 293 ± 24 (71 ± 637) |

Parameters are for "any drug effects". The threshold to determine times to onset and offset was set at 10% of the maximal possible response. Values are mean ± SEM (range). N=24; [α]N=18; [β]N=22 ; [γ]N=23

**Table S5. Mean values and statistics for the acute subjective effects of MDMA, S-MDMA, R-MDMA and placebo on the 5 Dimensions of Altered States of Consciousness (5D-ASC) Scale**

| | | Placebo mean ± SEM | 125 mg R-MDMA mean ± SEM | 250 mg R-MDMA mean ± SEM | 125 mg MDMA mean ± SEM | 125 mg S-MDMA mean ± SEM | $F_{4,92}$ | P= | Pla - R-MDMA (125 mg) | Pla - R-MDMA (250 mg) | Pla - MDMA | Pla - S-MDMA | R-MDMA (125 mg) - R-MDMA (250 mg) | R-MDMA (125 mg) - MDMA | R-MDMA (125 mg) - S-MDMA | R-MDMA (250 mg) - MDMA | R-MDMA (250 mg) - S-MDMA | MDMA - S-MDMA |
|---|---|---|---|---|---|---|---|---|---|---|---|---|---|---|---|---|---|---|
| 5 Dimensions of Altered States of Consciousness (5D-ASC) Scale | | | | | | | | | | | | | | | | | | |
| 5D-ASC total Score | % score | 0.6 ± 0.4 | 7.3 ± 1.8 | 17 ± 3.2 | 14 ± 3.3 | 17 ± 3.2 | 17.32 | <0.001 | * | *** | *** | *** | ** | * | ** | NS | NS | NS |
| 3D-ASC total Score | % score | 0.3 ± 0.3 | 6.4 ± 1.8 | 15 ± 3.3 | 14 ± 3.6 | 17 ± 3.7 | 15.04 | <0.001 | NS | *** | *** | *** | * | * | *** | NS | NS | NS |
| Oceanic boundlessness | % score | 0.5 ± 0.4 | 9.4 ± 2.9 | 19 ± 4.2 | 22 ± 5.1 | 23 ± 5.1 | 14.89 | <0.001 | NS | *** | *** | *** | * | ** | ** | NS | NS | NS |
| Anxious ego-dissolution | % score | 0.1 ± 0.0 | 2.8 ± 0.8 | 7.5 ± 1.9 | 5.8 ± 2.2 | 10 ± 3.1 | 6.94 | <0.001 | NS | ** | NS | *** | NS | NS | ** | NS | NS | NS |
| Visionary restructuralization | % score | 0.3 ± 0.3 | 6.0 ± 2.1 | 17 ± 4.7 | 12 ± 3.9 | 15 ± 4.2 | 9.30 | <0.001 | NS | *** | ** | *** | ** | NS | * | NS | NS | NS |
| Auditory alterations | % score | 0.0 ± 0.0 | 1.5 ± 0.7 | 8.2 ± 2.8 | 5.0 ± 2.5 | 5.5 ± 2.1 | 4.64 | 0.002 | NS | ** | NS | NS | * | NS | NS | NS | NS | NS |
| Reductions of vigilance | % score | 3.1 ± 1.5 | 21 ± 4.8 | 38 ± 6.3 | 26 ± 4.8 | 29 ± 4.2 | 16.38 | <0.001 | ** | *** | *** | *** | ** | NS | NS | NS | NS | NS |
| Experience of unity | % score | 0.6 ± 0.6 | 6.2 ± 2.9 | 19 ± 6.3 | 18 ± 6.5 | 21 ± 6.4 | 7.33 | <0.001 | NS | ** | ** | *** | NS | NS | * | NS | NS | NS |
| Spiritual experience | % score | 0.0 ± 0.0 | 3.6 ± 3.4 | 5.1 ± 3.0 | 8.2 ± 4.5 | 7.6 ± 4.2 | 2.57 | 0.043 | NS | NS | * | NS | NS | NS | NS | NS | NS | NS |
| Blissful state | % score | 1.2 ± 1.0 | 17 ± 5.0 | 28 ± 5.9 | 38 ± 7.1 | 35 ± 7.4 | 12.04 | <0.001 | NS | *** | *** | *** | NS | ** | * | NS | NS | NS |
| Insightfulness | % score | 0.2 ± 0.2 | 10 ± 4.2 | 17 ± 5.3 | 18 ± 5.7 | 16 ± 4.9 | 5.41 | <0.001 | NS | ** | ** | ** | NS | NS | NS | NS | NS | NS |
| Disembodiment | % score | 0.1 ± 0.1 | 4.6 ± 2.7 | 15 ± 5.0 | 12 ± 4.5 | 12 ± 3.9 | 4.82 | 0.001 | NS | ** | * | * | NS | NS | NS | NS | NS | NS |
| Impaired control and cognition | % score | 0.1 ± 0.1 | 4.4 ±1.2 | 13 ± 3.4 | 9.1 ± 3.2 | 15 ± 4.4 | 7.56 | <0.001 | NS | ** | * | *** | NS | NS | ** | NS | NS | NS |
| Anxiety | % score | 0.0 ± 0.0 | 1.3 ± 0.9 | 1.6 ± 0.7 | 2.5 ± 1.4 | 4.9 ± 2.3 | 2.41 | NS | - | - | - | - | - | - | - | - | - | - |
| Complex imagery | % score | 0.2 ± 0.1 | 8.3 ± 3.5 | 26 ± 7.0 | 17 ± 5.8 | 20 ± 6.3 | 7.42 | <0.001 | NS | *** | * | ** | * | NS | NS | NS | NS | NS |
| Elementary imagery | % score | 0.0 ± 0.0 | 5.6 ± 2.7 | 16 ± 6.1 | 9.4 ± 4.1 | 13 ± 4.9 | 3.28 | 0.015 | NS | * | NS | NS | NS | NS | NS | NS | NS | NS |
| Audio-visual synesthesia | % score | 0.0 ± 0.0 | 4.6 ± 2.2 | 12 ± 4.9 | 8.7 ± 3.9 | 15 ± 5.2 | 4.83 | 0.001 | NS | * | NS | ** | NS | NS | NS | NS | NS | NS |
| Changed meaning of percepts | % score | 0.3 ± 0.3 | 5.3 ± 2.8 | 15 ± 4.7 | 14 ± 4.9 | 14 ± 3.7 | 5.77 | <0.001 | NS | ** | ** | ** | NS | NS | NS | NS | NS | NS |

(*)P<0.1, *P<0.05, **P<0.01, ***P<0.001; NS, not significant N=24

**Table S6. Mean values and statistics for the acute subjective effects of MDMA, S-MDMA, R-MDMA and placebo on the Psychedelic Experience Questionnaire (PES48)**

| | | Placebo mean ± SEM | 125 mg R-MDMA mean ± SEM | 250 mg R-MDMA mean ± SEM | 125 mg MDMA mean ± SEM | 125 mg S-MDMA mean ± SEM | $F_{4,92}$ | P= | Pla - R-MDMA (125 mg) | Pla - R-MDMA (250 mg) | Pla - MDMA | Pla - S-MDMA | R-MDMA (125 mg) - R-MDMA (250 mg) | R-MDMA (125 mg) - MDMA | R-MDMA (125 mg) - S-MDMA | R-MDMA (250 mg) - MDMA | R-MDMA (250 mg) - S-MDMA | MDMA - S-MDMA |
|---|---|---|---|---|---|---|---|---|---|---|---|---|---|---|---|---|---|---|
| Mystical Experiences Questionnaire (MEQ30) | | | | | | | | | | | | | | | | | | |
| Mystical | % score | 0.6 ± 0.5 | 7.2 ± 3.1 | 15 ± 4.5 | 16 ± 4.6 | 14 ± 4.5 | 7.91 | <0.001 | NS | *** | *** | *** | NS | NS | NS | NS | NS | NS |
| Positive mood | % score | 2.4 ± 1.7 | 16 ± 3.8 | 30 ± 4.1 | 38 ± 4.7 | 38 ± 4.9 | 23.71 | <0.001 | * | *** | *** | *** | * | *** | *** | NS | NS | NS |
| Transcendence of time/space | % score | 1.5 ± 1.0 | 11 ± 2.4 | 25 ± 5.0 | 24 ± 4.7 | 25 ± 4.6 | 16.23 | <0.001 | NS | *** | *** | *** | ** | ** | ** | NS | NS | NS |
| Ineffability | % score | 1.1 ± 0.9 | 17 ± 4.3 | 34 ± 5.5 | 36 ± 6.3 | 48 ± 6.4 | 23.19 | <0.001 | * | *** | *** | *** | * | ** | *** | NS | NS | NS |
| MEQ30 total score | % score | 1.2 ± 0.9 | 11 ± 4.3 | 22 ± 4.0 | 24 ± 4.4 | 24 ± 4.2 | 20.37 | <0.001 | * | *** | *** | *** | ** | *** | *** | NS | NS | NS |
| Additional subscales on the Mystical Experience Questionnaire (MEQ40) | | | | | | | | | | | | | | | | | | |
| Paradoxicality | % score | 0.7 ± 0.5 | 1.7 ± 0.9 | 17 ± 4.3 | 12 ± 4.1 | 11 ± 2.8 | 9.08 | <0.001 | NS | *** | ** | * | *** | * | NS | NS | NS | NS |
| Connectedness | % score | 1.7 ± 1.2 | 20 ± 4.2 | 34 ± 4.7 | 34 ± 4.7 | 33 ± 5.6 | 16.87 | <0.001 | ** | *** | *** | *** | * | * | NS | NS | NS | NS |
| Additional subscales on the Psychedelic Experience Questionnaire/Scale (PES48) | | | | | | | | | | | | | | | | | | |
| Visual Experience | % score | 0.0 ± 0.0 | 2.2 ± 1.2 | 8.6 ± 3.9 | 2.5 ± 1.6 | 7.2 ± 3.3 | 3.52 | 0.01 | NS | * | NS | NS | NS | NS | NS | NS | NS | NS |
| Distressing Experience | % score | 0.5 ± 0.5 | 3.7 ± 1.9 | 10 ± 3.4 | 2.7 ± 0.9 | 7.5 ± 2.6 | 3.66 | 0.008 | NS | ** | NS | NS | NS | NS | NS | NS | NS | NS |
| Mystical Experiences Questionnaire (MEQ43) | | | | | | | | | | | | | | | | | | |
| Internal unity | % score | 0.3 ± 0.2 | 6.9 ± 2.9 | 15 ± 4.5 | 16 ± 4.3 | 13 ± 4.2 | 8.08 | <0.001 | NS | *** | *** | ** | NS | * | NS | NS | NS | NS |
| External unity | % score | 0.6 ± 0.6 | 3.6 ± 1.5 | 14 ± 4.5 | 14 ± 4.4 | 16 ± 4.7 | 8.35 | <0.001 | NS | ** | ** | *** | * | * | ** | NS | NS | NS |
| Sacredness | % score | 0.4 ± 0.4 | 6.3 ± 3.0 | 15 ± 3.6 | 13 ± 4.0 | 16 ± 4.2 | 8.66 | <0.001 | NS | *** | ** | *** | NS | NS | * | NS | NS | NS |
| Noetic quality | % score | 1.5 ± 1.3 | 14 ± 4.9 | 20 ± 5.6 | 16 ± 4.8 | 17 ± 4.5 | 5.94 | <0.001 | * | *** | ** | ** | NS | NS | NS | NS | NS | NS |
| Deeply felt positive mood | % score | 2.0 ± 1.4 | 18 ± 4.1 | 29 ± 4.6 | 41 ± 5.2 | 40 ± 5.3 | 21.89 | <0.001 | * | *** | *** | *** | NS | *** | *** | NS | NS | NS |
| Transcendence of time/space | % score | 1.6 ± 1.2 | 8.6 ± 2.0 | 24 ± 5.0 | 22 ± 4.9 | 21 ± 4.5 | 14.58 | <0.001 | NS | *** | *** | *** | *** | ** | ** | NS | NS | NS |
| Ineffability | % score | 1.0 ± 0.7 | 11 ± 2.9 | 29 ± 4.7 | 26 ± 5.2 | 32 ± 4.3 | 18.68 | <0.001 | NS | *** | *** | *** | *** | ** | *** | NS | NS | NS |

*P<0.05, **P<0.01, ***P<0.001; NS, not significant N=24

**Table S7. Mean values and statistics for the acute subjective effects of MDMA, S-MDMA, R-MDMA, and placebo on the Adjective Mood Rating Scale (AMRS)**

| | | Placebo | 125 mg R-MDMA | 250 mg R-MDMA | 125 mg MDMA | 125 mg S-MDMA | $F_{4,92}$ | P= | Pla - R-MDMA (125 mg) | Pla - R-MDMA (250 mg) | Pla - MDMA | Pla - S-MDMA | R-MDMA (125 mg) - R-MDMA (250 mg) | R-MDMA (125 mg) - MDMA | R-MDMA (125 mg) - S-MDMA | R-MDMA (250 mg) - MDMA | R-MDMA (250 mg) - S-MDMA | MDMA - S-MDMA |
|---|---|---|---|---|---|---|---|---|---|---|---|---|---|---|---|---|---|---|
| | | mean ± SEM | mean ± SEM | mean ± SEM | mean ± SEM | mean ± SEM | | | | | | | | | | | | |
| Adjective Mood Rating Scale (AMRS, score) | | | | | | | | | | | | | | | | | | |
| General well-being | $\Delta E_{max}$ | -0.0 ± 0.5 | 1.6 ± 1.0 | 2.9 ± 1.0 | 3.5 ± 1.1 | 4.7 ± 0.9 | 4.40 | 0.003 | NS | NS | * | ** | NS | NS | NS | NS | NS | NS |
| Emotional excitation | $\Delta E_{max}$ | -0.2 ± 0.7 | 1.4 ± 0.7 | 2.2 ± 0.8 | 3.3 ± 0.8 | 5.7 ± 0.7 | 10.56 | <0.001 | NS | NS | ** | *** | NS | NS | *** | NS | ** | NS |
| Extraversion | $\Delta E_{max}$ | 0.2 ± 0.4 | 1.3 ± 0.7 | 2.4 ± 0.6 | 4.0 ± 0.6 | 4.5 ± 0.6 | 11.52 | <0.001 | NS | * | *** | *** | NS | ** | *** | NS | NS | NS |
| Introversion | $\Delta E_{max}$ | 0.5 ± 0.3 | 2.2 ± 0.5 | 3.0 ± 0.6 | 1.4 ± 0.3 | 2.5 ± 0.4 | 7.11 | <0.001 | * | *** | NS | ** | NS | NS | NS | * | NS | NS |
| Activity | $\Delta E_{max}$ | 0.7 ± 0.4 | 1.6 ± 0.5 | 1.6 ± 0.7 | 1.8 ± 0.6 | 2.7 ± 0.6 | 2.28 | NS | - | - | - | - | - | - | - | - | - | - |
| Inactivity | $\Delta E_{max}$ | 3.4 ± 0.8 | 7.8 ± 1.8 | 12 ± 1.8 | 9.7 ± 1.4 | 11 ± 1.3 | 8.69 | <0.001 | NS | *** | ** | *** | NS | NS | NS | NS | NS | NS |
| Concentration | $\Delta E_{max}$ | 1.0 ± 0.3 | 1.0 ± 0.6 | 0.4 ± 0.4 | 0.9 ± 0.4 | 1.3 ± 0.7 | 0.50 | NS | - | - | - | - | - | - | - | - | - | - |
| Anxiety | $\Delta E_{max}$ | -0.3 ± 0.2 | 0.5 ± 0.3 | 0.4 ± 0.2 | 0.2 ± 0.2 | 0.9 ± 0.2 | 3.74 | 0.007 | NS | NS | NS | ** | NS | NS | NS | NS | NS | NS |

*P<0.05, **P<0.01, ***P<0.001; NS, not significant; $\Delta E_{max}$, maximal effect difference from baseline; $\Delta E_{min}$, minimal effect difference from baseline; N=24

**Table S8. Acute adverse drug effects after administration of MDMA, S-MDMA, R-MDMA and placebo**

| | Placebo | | | | 125 mg R-MDMA | | | | 250 mg R-MDMA | | | | 125 mg MDMA | | | | 125 mg S-MDMA | | | |
|---|---|---|---|---|---|---|---|---|---|---|---|---|---|---|---|---|---|---|---|---|
| | 0h | 0-9h | 9-24h | 24-72h | 0h | 0-9h | 9-24h | 24-72h | 0h | 0-9h | 9-24h | 24-72h | 0h | 0-9h | 9-24h | 24-72h | 0h | 0-9h | 9-24h | 24-72h |
| Fatigue | 14 | 13 | 10 | 10 | 16 | 18 | 16 | 13 | 13 | 21 | 19 | 12 | 10 | 20 | 18 | 14 | 16 | 22 | 21 | 13 |
| Headache | 3 | 5 | 6 | 6 | 1 | 13 | 11 | 14 | 4 | 15 | 15 | 11 | 3 | 9 | 12 | 11 | 4 | 16 | 11 | 11 |
| Lethargy | 0 | 5 | 2 | 4 | 1 | 13 | 9 | 11 | 0 | 15 | 12 | 9 | 2 | 14 | 9 | 9 | 2 | 17 | 14 | 11 |
| Decreased appetite | 1 | 0 | 1 | 4 | 0 | 12 | 9 | 8 | 1 | 14 | 11 | 7 | 2 | 14 | 8 | 6 | 1 | 16 | 10 | 7 |
| Feeling dull | 1 | 1 | 2 | 2 | 0 | 11 | 10 | 9 | 1 | 11 | 12 | 7 | 1 | 10 | 7 | 5 | 2 | 12 | 13 | 10 |
| Lack of concentration | 2 | 1 | 1 | 4 | 0 | 10 | 5 | 6 | 0 | 14 | 10 | 6 | 1 | 9 | 7 | 5 | 2 | 17 | 9 | 12 |
| Easily exhausted | 0 | 2 | 2 | 2 | 0 | 10 | 8 | 8 | 0 | 12 | 12 | 8 | 0 | 10 | 4 | 7 | 0 | 11 | 12 | 10 |
| Bruxism | 0 | 1 | 0 | 2 | 0 | 8 | 4 | 2 | 0 | 12 | 6 | 2 | 1 | 16 | 11 | 5 | 0 | 18 | 10 | 10 |
| Feeling of weakness | 0 | 2 | 1 | 3 | 0 | 8 | 4 | 8 | 1 | 11 | 13 | 7 | 0 | 6 | 5 | 5 | 0 | 9 | 13 | 11 |
| Hypersomnia | 0 | 3 | 1 | 3 | 0 | 9 | 6 | 4 | 1 | 13 | 9 | 5 | 0 | 7 | 6 | 3 | 0 | 12 | 12 | 9 |
| Dry mouth | 0 | 1 | 1 | 1 | 0 | 11 | 7 | 3 | 0 | 10 | 7 | 3 | 0 | 13 | 6 | 4 | 0 | 13 | 8 | 6 |
| Uneasiness | 2 | 1 | 1 | 2 | 1 | 8 | 3 | 5 | 0 | 14 | 6 | 4 | 0 | 8 | 2 | 3 | 1 | 11 | 5 | 12 |
| Tension | 3 | 1 | 0 | 2 | 1 | 7 | 2 | 2 | 2 | 11 | 3 | 3 | 1 | 4 | 2 | 2 | 1 | 10 | 3 | 9 |
| Job-related or personal worries | 4 | 1 | 2 | 2 | 3 | 4 | 3 | 3 | 1 | 7 | 4 | 3 | 3 | 4 | 1 | 4 | 2 | 6 | 3 | 6 |
| Dizziness | 0 | 1 | 0 | 0 | 0 | 7 | 4 | 4 | 0 | 8 | 5 | 4 | 0 | 5 | 3 | 2 | 0 | 11 | 5 | 6 |
| Heavy legs | 0 | 0 | 1 | 1 | 0 | 5 | 5 | 5 | 1 | 8 | 4 | 5 | 1 | 6 | 2 | 2 | 2 | 6 | 4 | 5 |
| Memory impairment | 1 | 0 | 2 | 2 | 0 | 5 | 3 | 3 | 1 | 6 | 3 | 6 | 0 | 3 | 4 | 2 | 1 | 6 | 5 | 8 |
| Obsessive rumination | 2 | 0 | 1 | 2 | 1 | 5 | 4 | 2 | 1 | 9 | 4 | 5 | 0 | 5 | 1 | 5 | 0 | 3 | 4 | 7 |
| Hyperhidrosis | 0 | 0 | 0 | 0 | 0 | 6 | 2 | 2 | 0 | 9 | 5 | 3 | 0 | 7 | 4 | 3 | 0 | 13 | 4 | 2 |
| Nausea | 0 | 1 | 0 | 1 | 1 | 5 | 5 | 4 | 0 | 8 | 7 | 6 | 0 | 5 | 2 | 2 | 0 | 5 | 4 | 3 |
| Vertigo | 0 | 1 | 1 | 0 | 0 | 4 | 3 | 2 | 0 | 11 | 3 | 4 | 0 | 7 | 3 | 2 | 0 | 8 | 3 | 6 |
| Crying | 0 | 0 | 0 | 3 | 0 | 5 | 3 | 5 | 1 | 4 | 4 | 6 | 0 | 5 | 3 | 5 | 0 | 2 | 3 | 8 |
| Insomnia | 2 | 0 | 1 | 2 | 0 | 1 | 5 | 5 | 0 | 4 | 5 | 4 | 0 | 3 | 5 | 4 | 1 | 2 | 1 | 8 |
| Micturition urgency | 0 | 4 | 2 | 0 | 1 | 7 | 1 | 2 | 0 | 9 | 1 | 1 | 0 | 7 | 2 | 1 | 1 | 9 | 3 | 1 |
| Hypersensitiviy to cold | 0 | 0 | 0 | 1 | 0 | 6 | 5 | 1 | 0 | 6 | 6 | 2 | 0 | 8 | 2 | 2 | 0 | 4 | 1 | 3 |
| Heart palpitations | 0 | 2 | 0 | 0 | 0 | 5 | 2 | 2 | 0 | 9 | 2 | 1 | 0 | 4 | 2 | 0 | 1 | 8 | 1 | 7 |
| Chills | 0 | 1 | 0 | 0 | 0 | 4 | 4 | 2 | 0 | 8 | 4 | 1 | 0 | 9 | 1 | 1 | 1 | 5 | 1 | 2 |
| Restlessness | 0 | 1 | 0 | 0 | 0 | 3 | 3 | 2 | 1 | 8 | 3 | 2 | 0 | 6 | 0 | 1 | 0 | 9 | 1 | 3 |
| Neck pain | 1 | 0 | 1 | 0 | 2 | 3 | 3 | 4 | 3 | 2 | 3 | 4 | 0 | 1 | 2 | 1 | 1 | 2 | 1 | 6 |
| Abnormal dreams | 1 | 1 | 1 | 1 | 0 | 0 | 1 | 4 | 2 | 1 | 1 | 3 | 0 | 2 | 2 | 4 | 1 | 3 | 2 | 7 |
| Hot flush | 0 | 0 | 0 | 0 | 0 | 4 | 1 | 1 | 0 | 10 | 2 | 1 | 0 | 6 | 1 | 1 | 0 | 8 | 1 | 1 |
| Peripheral coldness | 0 | 1 | 0 | 1 | 1 | 2 | 3 | 2 | 0 | 3 | 2 | 2 | 1 | 5 | 0 | 0 | 2 | 4 | 1 | 2 |
| Negative thoughts | 1 | 0 | 0 | 0 | 0 | 4 | 1 | 1 | 0 | 4 | 4 | 1 | 0 | 2 | 1 | 3 | 0 | 2 | 2 | 5 |
| Sensory processing sensitivity | 0 | 0 | 0 | 0 | 0 | 2 | 5 | 3 | 0 | 0 | 2 | 2 | 0 | 2 | 1 | 3 | 1 | 1 | 4 | 4 |
| Abdominal distension | 0 | 0 | 0 | 0 | 0 | 3 | 4 | 4 | 1 | 7 | 2 | 1 | 0 | 2 | 1 | 0 | 0 | 4 | 1 | 0 |
| Tremor | 0 | 0 | 0 | 0 | 0 | 3 | 1 | 1 | 0 | 6 | 0 | 0 | 0 | 5 | 0 | 1 | 0 | 8 | 1 | 4 |
| Abdominal pain | 0 | 1 | 1 | 0 | 0 | 2 | 4 | 5 | 1 | 1 | 2 | 1 | 0 | 3 | 3 | 2 | 0 | 1 | 0 | 1 |
| Discomfort | 0 | 0 | 0 | 0 | 1 | 3 | 4 | 2 | 0 | 3 | 2 | 1 | 0 | 0 | 0 | 2 | 0 | 3 | 1 | 6 |
| Irritability | 0 | 0 | 0 | 1 | 0 | 1 | 2 | 3 | 0 | 1 | 1 | 2 | 0 | 2 | 0 | 4 | 0 | 0 | 3 | 6 |
| Dysphagia | 1 | 1 | 2 | 2 | 0 | 1 | 2 | 2 | 0 | 1 | 2 | 1 | 0 | 0 | 1 | 1 | 1 | 3 | 2 | 2 |

Data indicate number of subjects reporting an effect among a total of 24 subjects.

**Table S9. Drug dose identification after session and after the study**

| | Placebo | | R-MDMA 125 mg | | R-MDMA 250 mg | | MDMA 125 mg | | S-MDMA 125 mg | |
|---|---|---|---|---|---|---|---|---|---|---|
| | after Session | after Study | after Session | after Study | after Session | after Study | after Session | after Study | after Session | after Study |
| correctly identified | 83% | 92% | 58% | 71% | 42% | 33% | 25% | 29% | 21% | 25% |
| missclassified as placebo | | | 21% | 4% | 0% | 0% | 0% | 0% | 4% | 4% |
| missclassified as R-MDMA 125 mg | 17% | 8% | | | 33% | 4% | 21% | 8% | 29% | 8% |
| missclassified as R-MDMA 250 mg | 0% | 0% | 8% | 8% | | | 25% | 33% | 13% | 25% |
| missclassified as MDMA 125 mg | 0% | 0% | 8% | 8% | 12.5% | 25% | | | 33% | 38% |
| missclassified as S-MDMA 125 mg | 0% | 0% | 4% | 8% | 12.5% | 38% | 29% | 29% | | |

after session = 24h after substance administration; after study = at end of study visit

*Consort flow chart*

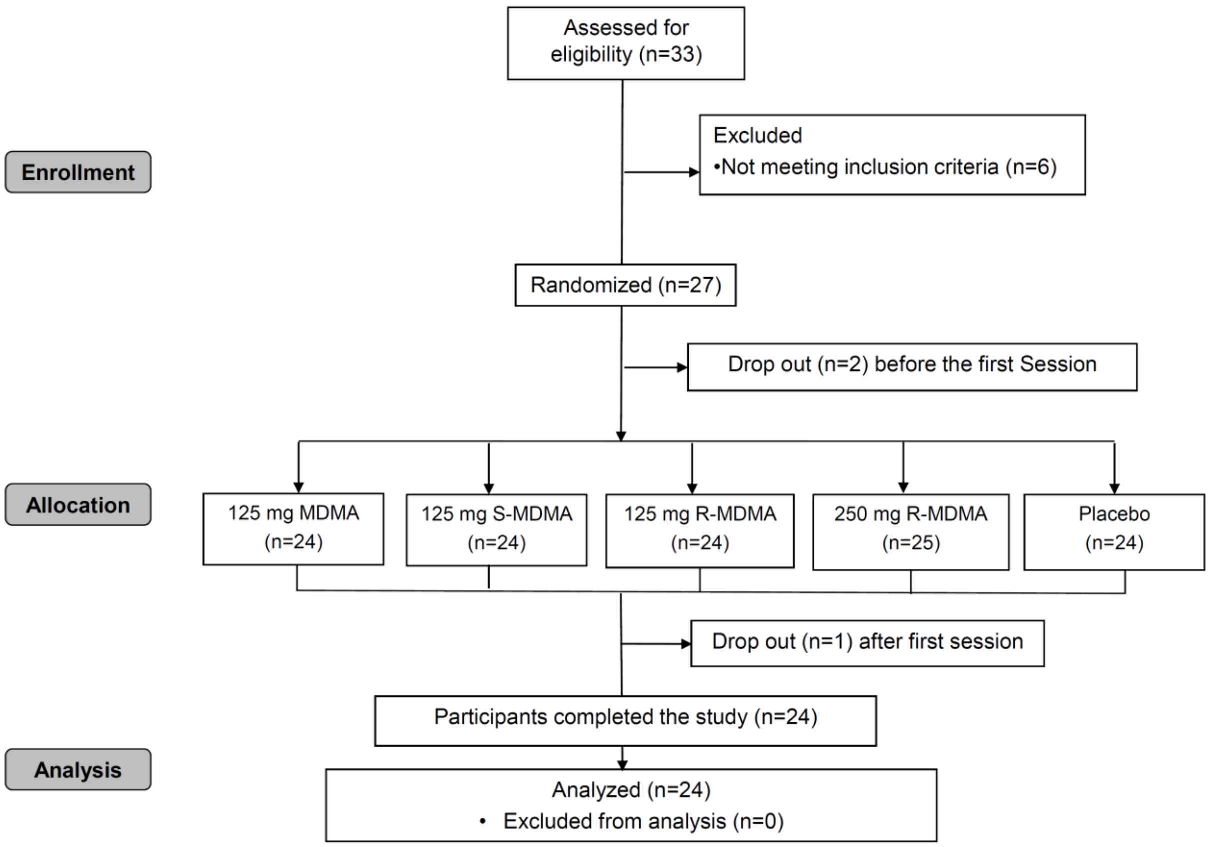

## References


1 Holze F, Vizeli P, Muller F, Ley L, Duerig R, Varghese N, et al. Distinct acute effects of LSD, MDMA, and D-amphetamine in healthy subjects. Neuropsychopharmacology. 2020;45:462-71.

2 Schmid Y, Enzler F, Gasser P, Grouzmann E, Preller KH, Vollenweider FX, et al. Acute effects of lysergic acid diethylamide in healthy subjects. Biol Psychiatry. 2015;78:544-53.

3 Hysek CM, Schmid Y, Simmler LD, Domes G, Heinrichs M, Eisenegger C, et al. MDMA enhances emotional empathy and prosocial behavior. Soc Cogn Affect Neurosci. 2014;9:1645-52.

4 Hysek CM, Liechti ME. Effects of MDMA alone and after pretreatement with reboxetine, duloxetine, clonidine, carvedilol, and doxazosin on pupillary light reflex. Psychopharmacology (Berl). 2012;224:363-76.

5 Straumann I, Ley L, Holze F, Becker AM, Klaiber A, Wey K, et al. Acute effects of MDMA and LSD co-administration in a double-blind placebo-controlled study in healthy participants. Neuropsychopharmacology. 2023;48:1840-48.

6 Ley L, Holze F, Arikci D, Becker AM, Straumann I, Klaiber A, et al. Comparative acute effects of mescaline, lysergic acid diethylamide, and psilocybin in a randomized, double-blind, placebo-controlled cross-over study in healthy participants. Neuropsychopharmacology. 2023;48:1659-67.

7 Holze F, Ley L, Muller F, Becker AM, Straumann I, Vizeli P, et al. Direct comparison of the acute effects of lysergic acid diethylamide and psilocybin in a double-blind placebo-controlled study in healthy subjects. Neuropsychopharmacology. 2022;47:1180-87.

8 Tagliazucchi E, Roseman L, Kaelen M, Orban C, Muthukumaraswamy SD, Murphy K, et al. Increased global functional connectivity correlates with LSD-induced ego dissolution. Curr Biol. 2016;26:1043-50.

9 Liechti ME, Dolder PC, Schmid Y. Alterations in conciousness and mystical-type experiences after acute LSD in humans. Psychopharmacology. 2017;234:1499-510.

10 Holze F, Duthaler U, Vizeli P, Muller F, Borgwardt S, Liechti ME. Pharmacokinetics and subjective effects of a novel oral LSD formulation in healthy subjects. Br J Clin Pharmacol. 2019;85:1474-83.

11 Janke W, Debus G. Die Eigenschaftswörterliste. Hogrefe: Göttingen.; 1978.

12 Dittrich A. The standardized psychometric assessment of altered states of consciousness (ASCs) in humans. Pharmacopsychiatry. 1998;31 (Suppl 2):80-4.

13 Studerus E, Gamma A, Vollenweider FX. Psychometric evaluation of the altered states of consciousness rating scale (OAV). PLoS One. 2010;5:e12412.

14 Carhart-Harris RL, Kaelen M, Bolstridge M, Williams TM, Williams LT, Underwood R, et al. The paradoxical psychological effects of lysergic acid diethylamide (LSD). Psychol Med. 2016;46:1379-90.

15 Dolder PC, Schmid Y, Mueller F, Borgwardt S, Liechti ME. LSD acutely impairs fear recognition and enhances emotional empathy and sociality. Neuropsychopharmacology. 2016;41:2638-46.

16 Bershad AK, Schepers ST, Bremmer MP, Lee R, de Wit H. Acute subjective and behavioral effects of microdoses of lysergic acid diethylamide in healthy human volunteers. Biol Psychiatry. 2019;86:792-800.

17 Preller KH, Herdener M, Pokorny T, Planzer A, Kraehenmann R, Stämpfli P, et al. The fabric of meaning and subjective effects in LSD-induced states depend on serotonin 2A receptor activation  Curr Biol. 2017;27:451-57.

18 Schmid Y, Gasser P, Oehen P, Liechti ME. Acute subjective effects in LSD- and MDMA-assisted psychotherapy. J Psychopharmacol. 2021;35:362-74.

19 Roseman L, Nutt DJ, Carhart-Harris RL. Quality of acute psychedelic experience predicts therapeutic efficacy of psilocybin for treatment-resistant depression. Front Pharmacol. 2017;8:974.

20 Griffiths RR, Johnson MW, Carducci MA, Umbricht A, Richards WA, Richards BD, et al. Psilocybin produces substantial and sustained decreases in depression and anxiety

in patients with life-threatening cancer: a randomized double-blind trial. J Psychopharmacol. 2016;30:1181-97.
21 Holze F, Gasser P, Muller F, Dolder PC, Liechti ME. Lysergic acid diethylamide-assisted therapy in patients with anxiety with and without a life-threatening illness: a randomized, double-blind, placebo-controlled phase II study. Biol Psychiatry. 2023;93:215-23.
22 Stocker K, Hartmann M, Ley L, Becker AM, Holze F, Liechti ME. The revival of the psychedelic experience scale: revealing its extended-mystical, visual, and distressing experiential spectrum with LSD and psilocybin studies. J Psychopharmacol. 2024;38:80-100.
23 Griffiths RR, Richards WA, McCann U, Jesse R. Psilocybin can occasion mystical-type experiences having substantial and sustained personal meaning and spiritual significance. Psychopharmacology. 2006;187:268-83; discussion 84-92.
24 Barrett FS, Johnson MW, Griffiths RR. Validation of the revised Mystical Experience Questionnaire in experimental sessions with psilocybin. J Psychopharmacol. 2015;29:1182-90.
25 MacLean KA, Johnson MW, Griffiths RR. Mystical experiences occasioned by the hallucinogen psilocybin lead to increases in the personality domain of openness. J Psychopharmacol. 2011;25:1453-61.
26 Griffiths RR, Johnson MW, Richards WA, Richards BD, McCann U, Jesse R. Psilocybin occasioned mystical-type experiences: immediate and persisting dose-related effects. Psychopharmacology. 2011;218:649-65.
27 Griffiths R, Richards W, Johnson M, McCann U, Jesse R. Mystical-type experiences occasioned by psilocybin mediate the attribution of personal meaning and spiritual significance 14 months later. J Psychopharmacol. 2008;22:621-32.
28 Garcia-Romeu A, Griffiths RR, Johnson MW. Psilocybin-occasioned mystical experiences in the treatment of tobacco addiction. Curr Drug Abuse Rev. 2014;7:157-64.
29 Garcia-Romeu A, Davis AK, Erowid F, Erowid E, Griffiths RR, Johnson MW. Cessation and reduction in alcohol consumption and misuse after psychedelic use. J Psychopharmacol. 2019;33:1088-101.
30 Griffiths RR, Johnson MW, Richards WA, Richards BD, Jesse R, MacLean KA, et al. Psilocybin-occasioned mystical-type experience in combination with meditation and other spiritual practices produces enduring positive changes in psychological functioning and in trait measures of prosocial attitudes and behaviors. J Psychopharmacol. 2018;32:49-69.
31 Ross S, Bossis A, Guss J, Agin-Liebes G, Malone T, Cohen B, et al. Rapid and sustained symptom reduction following psilocybin treatment for anxiety and depression in patients with life-threatening cancer: a randomized controlled trial. J Psychopharmacol. 2016;30:1165-80.
32 Becker AM, Klaiber A, Holze F, Istampoulouoglou I, Duthaler U, Varghese N, et al. Ketanserin reverses the acute response to LSD in a randomized, double-blind, placebo-controlled, crossover study in healthy participants. Int J Neuropsychopharmacol. 2023;26:97-106.
33 Holze F, Vizeli P, Ley L, Muller F, Dolder P, Stocker M, et al. Acute dose-dependent effects of lysergic acid diethylamide in a double-blind placebo-controlled study in healthy subjects. Neuropsychopharmacology. 2021;46:537-44.
